# Brain Transcriptomic Response to Social Eavesdropping in Zebrafish (*Danio rerio*)

**DOI:** 10.1371/journal.pone.0145801

**Published:** 2015-12-29

**Authors:** João Sollari Lopes, Rodrigo Abril-de-Abreu, Rui F. Oliveira

**Affiliations:** 1 Instituto Gulbenkian de Ciência, Oeiras, Portugal; 2 ISPA—Instituto Universitário, Lisboa, Portugal; 3 Champalimaud Neuroscience Programme, Champalimaud Centre for the Unknown, Lisboa, Portugal; Radboud University Nijmegen, NETHERLANDS

## Abstract

Public information is widely available at low cost to animals living in social groups. For instance, bystanders may eavesdrop on signaling interactions between conspecifics and use it to adapt their subsequent behavior towards the observed individuals. This social eavesdropping ability is expected to require specialized mechanisms such as social attention, which selects social information available for learning. To begin exploring the genetic basis of social eavesdropping, we used a previously established attention paradigm in the lab to study the brain gene expression profile of male zebrafish (*Danio rerio*) in relation to the attention they paid towards conspecifics involved or not involved in agonistic interactions. Microarray gene chips were used to characterize their brain transcriptomes based on differential expression of single genes and gene sets. These analyses were complemented by promoter region-based techniques. Using data from both approaches, we further drafted protein interaction networks. Our results suggest that attentiveness towards conspecifics, whether interacting or not, activates pathways linked to neuronal plasticity and memory formation. The network analyses suggested that *fos* and *jun* are key players on this response, and that *npas4a*, *nr4a1* and *egr4* may also play an important role. Furthermore, specifically observing fighting interactions further triggered pathways associated to a change in the alertness status (*dnajb5*) and to other genes related to memory formation (*btg2*, *npas4b*), which suggests that the acquisition of eavesdropped information about social relationships activates specific processes on top of those already activated just by observing conspecifics.

## Introduction

Group living animals may extract information from signalling interactions between conspecifics and use it to adjust their subsequent behavior towards the observed individuals, without the costs of first-hand experience (aka social eavesdropping [1,2]). Thus, this ability to adjust behaviour to a dynamic social environment is expected to impact Darwinian fitness. As such, it has been proposed that group living may have selected for cognitive processes that promote the ability for animals to use social information [3–5]. These adaptive specializations in cognition may have evolved both at the level of learning mechanisms and at the level of input mechanisms [6] such as social attention, which detect social information available for learning. For instance, to eavesdrop on conspecific interactions an animal must first be able to detect, approach and attend the signalling conspecifics between a multitude of other social and non-social stimuli in order to select and extract relevant information. This suggests that tuning of attention towards social interactions should be an essential mechanism for successful social eavesdropping.

While social eavesdropping has been investigated at the behavioral level in several species [7–10], to our knowledge its neural mechanisms and implications at the brain gene expression level have never been studied. However, it is known that the input of specific social information is linked to changes in gene activation in the brain, which in turn influence subsequent behavioral outputs [11]. Moreover, different behaviors have been shown to be strongly associated with different brain gene expression profiles [12]. For example previous work in our lab using zebrafish, a highly social model organism [13] with a wide set of neurogenetic tools available [14], has shown that a social acute agonistic event, like the experience of winning or losing a fight, is enough to elicit massive changes to the brain gene expression profiles of the interacting fish (Oliveira et al, 2015). In the case of social eavesdropping, it should also be expected that a bystander to a third party interaction will present different brain gene expression profiles reflecting the process of information acquisition and also its attentional state towards the interacting conspecifics (i.e. actively eavesdropping or not).

We started exploring the genetic basis of social eavesdropping as a follow-up to a previous study in our lab that used a one-trial preference task in which a bystander male zebrafish could observe, without being observed: an agonistic interaction (fight) between two male conspecifics; two non-interacting male conspecifics; or an empty tank (socially isolated) [15]. The behavior of bystanders was used as a read out of attention, by using a combination of measures such as sustained proximity, body orientation and directional focus. The study revealed that zebrafish were more attentive towards interacting (i.e. fighting) than towards non-interacting pairs of conspecifics, with the exposure to fighting not affecting activity or stress levels of the bystanders [15]. This tuning of attention is expected to be an essential aspect for social eavesdropping, in this case on the dominance status of the opponents, without incurring in the costs associated with fighting, which has already been shown to occur in zebrafish [16].

In this study, we selected representative individuals from each of the three treatments described above according to their behavioral profile, and used microarray gene chips to study their brain transcriptome. Our main goal was to characterize distinctive transcriptomic profiles associated with social information acquisition and to identify candidate genes related to attentiveness in general and to social eavesdropping in particular. Our approach was based on differential expression of single genes and of gene sets relative to a reference group of socially isolated individuals. We complemented these analyses by considering the alignment of transcription factor (TF) motifs with the promoter region of differentially expressed (DE) genes. Finally, we used data from both approaches to draft a protein interaction network that may be used as a base to understand the mechanisms behind the resulting transcriptomes. This approach had the advantage of allowing us to analyze the social regulation of gene expression and its underlying biological processes in freely moving zebrafish, while in a social eavesdropping context.

## Materials and Methods

### Behavioral task and procedures

Adult male wild-type (AB) zebrafish (11 months old) bred and held at Instituto Gulbenkian de Ciência (IGC, Oeiras, Portugal) were used. All fish were kept in mixed sex groups in environmentally enriched stock tanks (gravel substrate, artificial plants and rocks). All procedures were reviewed by the Instituto Gulbenkian de Ciência Ethics Committee, and approved by the competent Portuguese authority (Direcção Geral de Alimentação e Veterinária, permit 008955).

**Fig 1:**
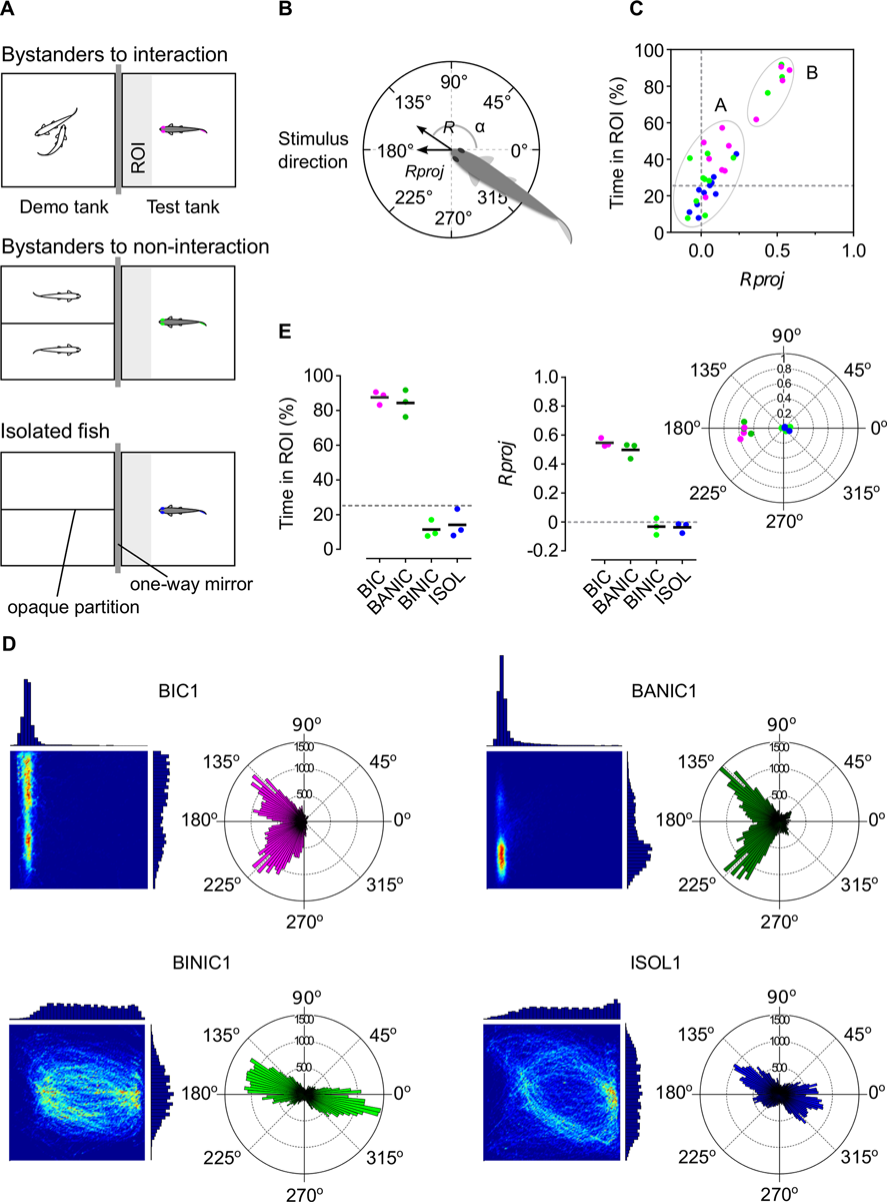
Behavioral paradigm and selected behavioral groups for transcriptomic analysis. (A) Schematics of experimental treatments bystanders to interacting conspecifics (magenta), bystanders to non-interacting conspecifics (lime); and isolated fish (blue). ROI in light grey. (B) Schematic of the subject fish’s mean resultant directional vector composed by the vector’s length *R*, mean angle α (0° opposite and 180° directed towards the stimulus) and *R* projected onto 180° (*Rproj*). (C) Clustering analysis of all focal fish from the experimental treatments using time spent in ROI and *Rproj*. Cluster A–strongly attentive profile; cluster B–weakly attentive profile. (D) Linear histograms and 2D heatmaps of time spent in each position of the arena (left), and polar directional histograms (right) of one individual per behavioral group (for complete set of samples, see S1 Fig). Heatmaps are scaled from maximum relative value (red) to minimum relative value (dark blue). Linear and polar histograms represented in arbitrary scale. (E) Scatter plots of selected fish from the four behavioral groups [bystanders to interacting conspecifics (BIC, magenta), bystanders attentive to non-interacting conspecifics (BANIC, green), bystanders inattentive to non-interacting conspecifcs (BINIC, lime), and isolated fish (ISOL, blue) for time in ROI (left), *Rproj* (center) and mean resultant directional vectors (right).

Thirty nine fish were previously subjected to one of three treatments (13 individuals per group) for 30 minutes (Fig 1A): (1) bystanders to a pair of interacting (i.e. fighting) male conspecifics; (2) bystanders to two non-interacting male conspecifics; and (3) socially isolated [15]. Each focal fish was subjected to a single test after an overnight baseline period of isolation in the corresponding test tank. Bystanders could visually observe through a one-way mirror the corresponding stimuli fish without being observed and no chemical communication was possible. Individual behavior was video recorded from a top-down view perspective and analyzed using a custom-made video tracking system that tracked the body position of each fish inside a defined region (arena) of the test tank (further details in [15]).

Immediately after the test, each focal fish was euthanized with an overdose of tricaine solution (MS222, Pharmaq; 500–1000 mg/L) and its spinal cord was sectioned. The brain was immediately removed and stored at -80°C in qiasol for posterior analysis. The selection of this sampling time was based on the fact that another study from our lab found massive changes in the brain transcriptome immediately after a 30 min agonistic interaction. Despite this early sampling time these changes comprised not only immediately early genes but also genes related to neural plasticity, immune function and epigenetic modifications (Oliveira et al., 2015).

Attentiveness of each focal fish was parameterized from its position in the arena and its body orientation (Fig 1B). From these values, a set of behavioral parameters were determined and analyzed at an individual and group level for each treatment: length (*R*) of the mean resultant directional vector (Fig 1B); projection of *R* (*Rproj*) onto the stimulus direction (180° line) (Fig 1B); total time spent in proximity to the stimulus in a defined region of interest (ROI, Fig 1A); mean speed in the ROI; and total distance covered in the arena.

### Defining behavioral profiles for transcriptomics analysis

In order to characterize different attentional profiles, we focused on the attentional behavioral parameters that revealed statistically significant differences with the reference group (isolated fish), namely, time in ROI and *Rproj* [15]. Using a partition around the medoids (PAM) method, we clustered all samples based on these two parameters. The number of clusters to use was defined by maximizing the average silhouette for all possible number of clusters (between 2 and 32). The PAM clustering used Euclidean distances with normalized values (i.e. values were subtracted by the variable's mean value and divided by the variable's mean absolute deviation), and was performed using the R [17] package “cluster”. Based on the PAM clustering analysis and similarities of spatial and directional behavioral patterns, we selected four representative groups, each composed of three sampled focal fish.

### Pre-processing of microarrays

RNA was extracted from the selected focal fishes’ brains using the RNeasy Lipid Tissue Mini kit (Qiagen) with some procedural modifications. In brief, samples were homogenized by vortex and added 20µl of chloroform. Incubation times were increased in order to maximize RNA recovery and in the end samples were diluted in 25µl of RNase-free water. RNA integrity was verified using Bioanalyzer prior to microarray gene array processing [18]. RNA was processed and used in Affymetrix zebrafish gene 1.1 ST array strips according to the manufacturer’s protocol. All microarray procedures were performed at the Gene Expression Unit of Instituto Gulbenkian de Ciência (IGC, Oeiras, Portugal). CEL files containing raw data were then processed and analyzed using R and Bioconductor packages [19]. These CEL files have been deposited at the NCBI Gene Expression Omnibus (GEO, waiting for accession number). Quality assurance of microarray data was assessed and all microarrays showed high quality data. The arrays were then pre-processed using the standard RMA normalization (see S1 Methods for details). The microarray dataset have been deposited in NCBI's Gene Expression Omnibus (GEO) repository and is accessible through GEO Series accession number GSE69719.

### Statistical analysis of microarray data

The selection of DE genes was performed considering an experimental design with the isolated group as a reference and using the three remaining groups one at a time. A linear model on log_2_ signal values with empirical Bayes correction to the variance (implemented in Bioconductor package “limma”) was used, and the *P*-values were adjusted for multiple testing using false discovery rates (FDR). The threshold for DE genes was set at FDR < 0.05 and fold-change > 2 or < 0.5. Using the pooled group of DE genes for the tested groups, a hierarchical cluster of both samples and genes was created.

Genes were annotated using Entrez IDs obtained primarily from the Bioconductor database, NCBI and biomart. A total of 21 224 genes were annotated, from which 20 944 had information on chromosome location. Over-represented analyses (ORA) were performed to assess if the DE genes of each behavior group were enriched in some gene sets. The gene sets considered were pathways from KEGG [20] and Wikipathway [21], terms from GO [22], and chromosome locations. The threshold for overrepresentation was set to *P*-value < 0.10. Because of the reduced number of DE genes obtained (see Results), ORA results should be interpreted with caution. Thus, we also performed gene set enrichment analyses (GSEA). Unlike ORA, GSEA uses the whole gene expression data instead of defining a list of strongly DE genes. There are many flavors of GSEA [23]; we applied the parametric competitive method Generally Applicable Gene-set [GAGE, [24]] which is adequate for small datasets and allows for analysis considering only up- or down-regulated genes, or both. The gene sets used were also from KEGG, Wikipathways, GO terms and chromosome locations, and the threshold was also set to *P*-value < 0.10. These analyses were performed using Bioconductor packages “biomaRt” and “reutils” (annotation), “GO.db”, “KEGG.db, “Catergory” and “GOstats” (ORA), and “gage” and “GSEABase” (GAGE).

### Promoter region analysis and transcription network

Promoter analyses identify TF motifs associated to up- or down-regulated genes. Since multiple TFs may have nearly identical motifs, the statistical findings are related to the motif itself and not to the TF where it came from. Nevertheless, for simplicity, we used the TF nomenclature to name the motifs. TF binding sites (motifs) were searched in upstream regions of the Zebrafish genome by calculating scores using Stubb 2.1 [25]. These scores were used to perform enrichment analysis using cis-Metalysis [26] by considering a set of DE genes identified for each behavior group. The algorithm used for these analyses is similar to the procedure by Sanogo and co-workers [27] and is detailed in the S1 Methods. In brief, genomic information was obtained from UCSC Genome Browser, to which Stubb was used to score motifs every 500 bp windows with a 250 bp shift. Non-redundant motifs from Jaspar Core Vertebrate database [28] were considered. Enrichment analyses were then performed for each motif and pair of motifs using cis-Metalysis (mode “flexible”). Using STRING 9.1 [29], we further constructed transcription networks considering *Homo sapiens* homologs of the list of DE genes and of enriched TF for each social treatment (required confidence for edges was set to score > 0.4). These networks were then analyzed regarding centralization, density, heterogeneity and structural correlation.

Analyses were performed using Stubb 2.1 and cis-Metalysis within a python pipeline (scripts available upon request). Network analyses were performed using STRING 9.1 and R package "sna".

## Results

### Behavioral profiles

The behavioral parameters that showed statistically significant differences to the reference group (isolated fish) were *Rproj* and time in ROI. In both of them we can define a chance level corresponding to no focus nor increased proximity towards the stimulus (i.e. *Rproj* = 0 and time in ROI = 25%; grey dashed lines in Fig 1A). Isolated fish were characterized by time in ROI = 22.67 ± 3.17% and *Rproj* = 0.041 ± 0.027 (mean ± s.e.m., n = 10), which were significantly lower than values for bystanders to interacting conspecifics (time in ROI = 55.05 ± 7.22%, *Rproj* = 0.25 ± 0.065, n = 11), but not lower than values for bystanders to non-interacting conspecifics (time in ROI = 41.58 ± 8.20%, *Rproj* = 0.14 ± 0.066, n = 12). Also, while bystanders to interacting conspecifics were almost all above chance level and isolated fish were around that level, bystanders to non-interacting conspecifics were composed by a majority of fish spread close to chance level and by some clearly above it. Considering two clusters (average silhouette of 0.72), two distinct groups were created (Fig 1C): cluster A with mean time in ROI = 82.50 ± 1.51% and mean *Rproj* = 0.50 ± 0.01, composed by four bystanders to interacting conspecifics and three bystanders to non-interacting conspecifics; and cluster B with mean time in ROI = 28.99 ± 0.52% and mean *Rproj* = 0.05 ± 0.003, composed by the remaining bystanders to interacting conspecifics, bystanders to non-interacting conspecifics and isolated fish. This result supported the existence of a strongly attentive profile (cluster A) composed by bystander fish that spent most of the time in close proximity to the stimulus and with a high directional focus towards it, and a weakly attentive profile (cluster B) composed by fish that showed little proximity and directional focus towards the stimulus.

Based on these profiles and on the matching of individual spatial and directional patterns (Fig 1D and S1 Fig), we created four sample groups of interest (three fish per group, Fig 1E): (1) highly attentive bystander fish selected from the bystanders to interacting conspecifics treatment and belonging to cluster A; (2) highly attentive bystanders from cluster A but selected from the bystanders to non-interacting conspecifics treatment; (3) inattentive fish selected from the bystanders to non-interacting conspecifics treatment and from cluster B; and (4) inattentive fish selected from the reference treatment (isolated fish). Interestingly, the high attentiveness towards the stimulus showed by the directionality of the sampled bystanders to interacting conspecifics and bystanders attentive to non-interacting conspecifics resulted from the collapsing of a bimodal distribution peaking at an approximate 45° angle deviation from the 180° direction which may be related to zebrafish eyes positioning and field of view (see S1 Fig).

### Brain transcriptome profiles

**Fig 2:**
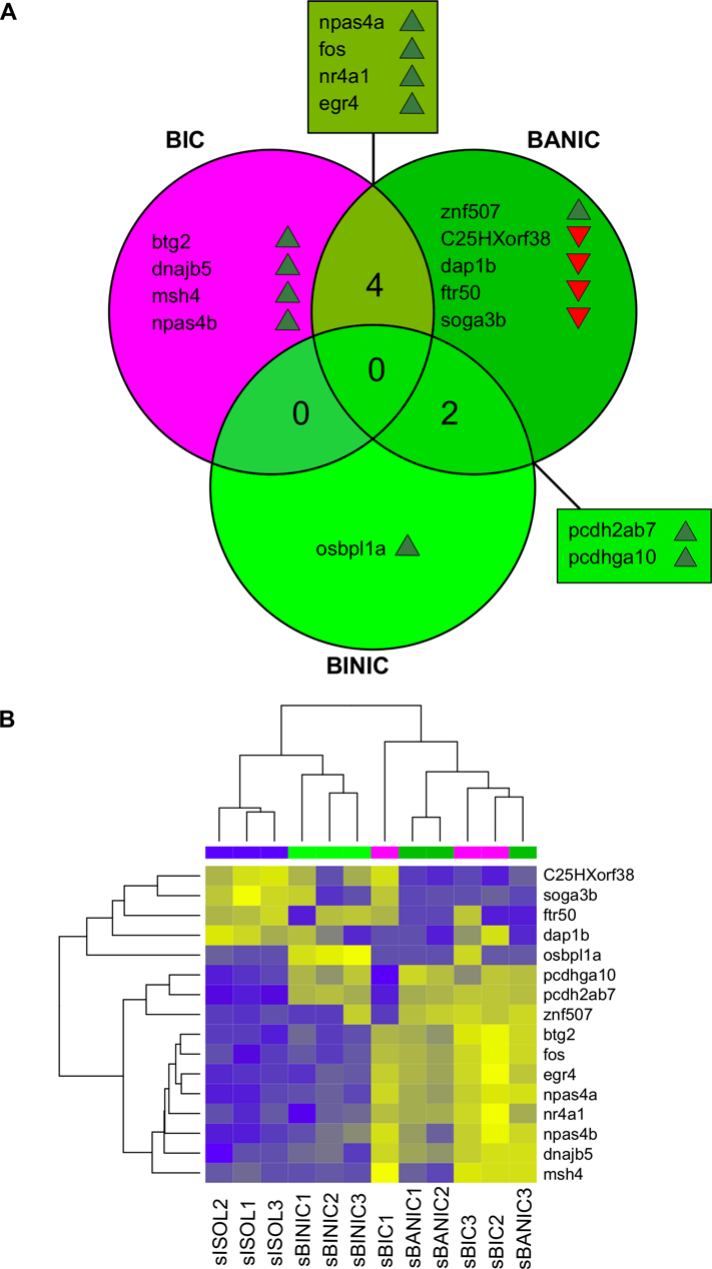
Changes in gene expression in the brain of bystander zebrafish. (A) Venn diagram showing DE genes between behavioral groups (BIC, bystanders to interacting conspecifics; BANIC, bystanders attentive to non-interacting conspecifics; and BINIC, bystanders inattentive to non-interacting conspecifics), and the reference group (ISOL, isolated fish) (up-regulated = ▴; down-regulated = ▾). (B) Hierarchical clustering of the individuals from each selected behavior group (columns) and of DE genes (lines). Heatmap represents normalized gene expression levels (blue = low expression, yellow = high-expression).

Comparing the whole-brain transcriptome of the reference group to the remaining groups revealed that 4 DE genes were only associated to bystanders to interacting conspecifcs, 5 DE genes were only associated to bystanders attentive to non-interacting conspecifics and 4 DE genes were associated to both. Only 1 DE gene was associated to bystanders inattentive to non-interacting conspecifics, and 2 DE genes were shared by bystanders attentive to non-interacting conspecifics and bystanders inattentive to non-interacting conspecifics (Fig 2A and S1–S3 Tables, see also Table 1 for a summary of their functions and respective references). All the DE genes associated to both bystanders to interacting conspecifics and bystanders attentive to non-interacting conspecifics were neuronal activity-dependent immediate early genes (i.e. *egr4*, *fos*, *npas4a*, and *nr4a1*) with a role in neural plasticity and brain activity. The DE genes associated only to bystanders to interacting conspecifics also included neuronal activity-dependent immediate early genes associated to neuronal plasticity (i.e. *btg2* and *npas4b*), and the late gene *dnajb5*, which has a role in stress regulation and in circadian neuronal circuit in *Drosophila*. DE genes associated to both bystanders attentive to non-interacting conspecifics and bystanders inattentive to non-interacting conspecifics code for protocadherin proteins (i.e. *pcdh2ab7* and *pcdhga10*), which play a role in self-recognition of individual neurons. As for the DE genes associated only to bystanders attentive to non-interacting conspecifics, they do not have a clear link to neuronal functions. The DE gene *osbpl1a*, unique to group bystanders inattentive to non-interacting conspecifics, also does not have a clear neuronal function.

**Table 1:** Summary of the functions of at least one DE gene or one enriched TF.

Function	DE genes or enriched TF motifs^a^
cell-cell communication	*pcdh2ab7* and *pcdhga10* [30]
cholesterol biosynthesis	*osbpl1a* [31]
circadian neuronal circuit	*dnajb5* [32], *fos* [33], and JUN::FOS [34]
development of nervous system	CDX2 [35], GATA2 [36], HNF1B [37], PDX1 [38], and TAL1 [39]
memory formation	*btg2* [40], *egr4* [41], *fos* [42], JUN [43], MEF2A^b^ [44], *npas4* ^b^ [45], SRF [46]
neuronal cells effect	E2F1[47], JUN::FOS [48], MYC [49], and REST [50]
response to cellular stress	*dnajb5* [32], JUN [51], JUN::FOS [52], MEF2A [53], MYC [54]
sensorial system	FOXQ1 [55], RFX2 [56]

DE, differentially expressed; TF, transcription factor

^a^DE gene is represented in italicized small caps, TF motif is represented in all caps

^b^role in fear memory

Hierarchical clustering of the samples indicated that the behavioral groups are well defined, although to a lesser extent between groups bystanders to interacting conspecifics and bystanders attentive to non-interacting conspecifics (Fig 2B). As observed in the behavioral profiles (Fig 1D and 1E), the gene expression profile of bystanders inattentive to non-interacting conspecifics was closer to isolated fish than to the remaining groups, and there seemed to be a pairing between bystanders attentive to non-interacting conspecifics and bystanders to interacting conspecifics. Hierarchical clustering of the genes created a well-defined subset of genes with a similar profile of expression across all the twelve individuals (*btg2*, *dnajb5*, *egr4*, *fos*, *msh4*, *npas4a*, *npas4b* and *nr4a1*).

Results for ORA (S4–S9 Tables) showed that DE genes shared by bystanders to interacting conspecifics and by bystanders attentive to non-interacting conspecifics were members of the MAPK signaling pathway (*fos* and *nr4a1*), had a metabolic and/or biosynthetic role and were located in the nucleus (*fos*, *nr4a1* and *npas4a*), and had a binding function (*egr4*, *fos*, *nr4a1* and *npas4a*). One DE gene unique to bystanders to interacting conspecifics (*dnajb5*) had a metabolic and/or biosynthetic role and a binding function; and 2 DE genes unique to bystanders attentive to non-interacting conspecifics (*pcdh2ab7* and *ftr50*) also had a binding function. Finally, there seemed to be an over-enrichment of DE genes located in chromosome 23 for bystanders to interacting conspecifics (*egr4* and *nr4a1*) and for bystanders attentive to non-interacting conspecifics (*egr4*, *nr4a1* and *soga3b*), and in chromosome 14 also for bystanders attentive to non-interacting conspecifics (*pcdh2ab7*, *pcdhga10* and *npas4a*) and for bystanders inattentive to non-interacting conspecifics (*pcdh2ab7*, *pcdhga10*).

The gene set enrichment GAGE results (S10–S15 Tables) showed that bystanders to interacting conspecifics and bystanders attentive to non-interacting conspecifics had distinct profiles of DE gene sets. Pathways enriched in bystanders to interacting conspecifics included “Phototransduction”, “Exercise-induced circadian regulation”, and “Cholesterol/Steroid biosynthesis”, which may be related to cortisol production, and various growth-related pathways, whereas bystanders attentive to non-interacting conspecifics and bystanders inattentive to non-interacting conspecifics were enriched in metabolism-based pathways and in one pathway each that was shared with bystanders to interacting conspecifics (“FGF signaling pathway” in bystanders attentive to non-interacting conspecifics and “Cholesterol/Steroid Biosynthesis” in bystanders inattentive to non-interacting conspecifics). Unsurprisingly, GO analyses of gene sets using biological process terms showed that all groups were enriched in transcription-related terms. However, bystanders to interacting conspecifics were also enriched in the generic term “response to stress” and in the term “lipid metabolic process”, which may be related to hormone production, whereas bystanders attentive to non-interacting conspecifics were enriched in the neurogenesis-related term “notch signaling pathway” and in terms related to visual and audio sensory systems. Interestingly, bystanders inattentive to non-interacting conspecifics were also enriched in genes linked to sensory organs development. Regarding GO terms of cellular compartments, bystanders to interacting conspecifics were enriched in terms related to cell-cell communication, while bystanders attentive to non-interacting conspecifics and bystanders inattentive to non-interacting conspecifics were enriched in the term “peroxisome”, which is related to metabolism and possibly to cholesterol biosynthesis. As for the analyses using GO terms of molecular functions, we also found that all behavioral groups were enriched in transcription-related terms. Moreover, bystanders to interacting conspecifics were also enriched in growth-related terms, in the fight-or-flight term “adrenergic receptor activity”, in the metabolism-related term “cytochrome-c oxidase activity” and in terms related to cell-cell signaling, whereas bystanders attentive to non-interacting conspecifics were further enriched in the term related to cell-cell signaling “voltage-gated potassium channel activity” and in term “photoreceptor activity”. Finally, regarding chromosome location, genes from chromosome 14 were enriched in all behavioral groups.

### Brain transcription network profiles

**Fig 3:**
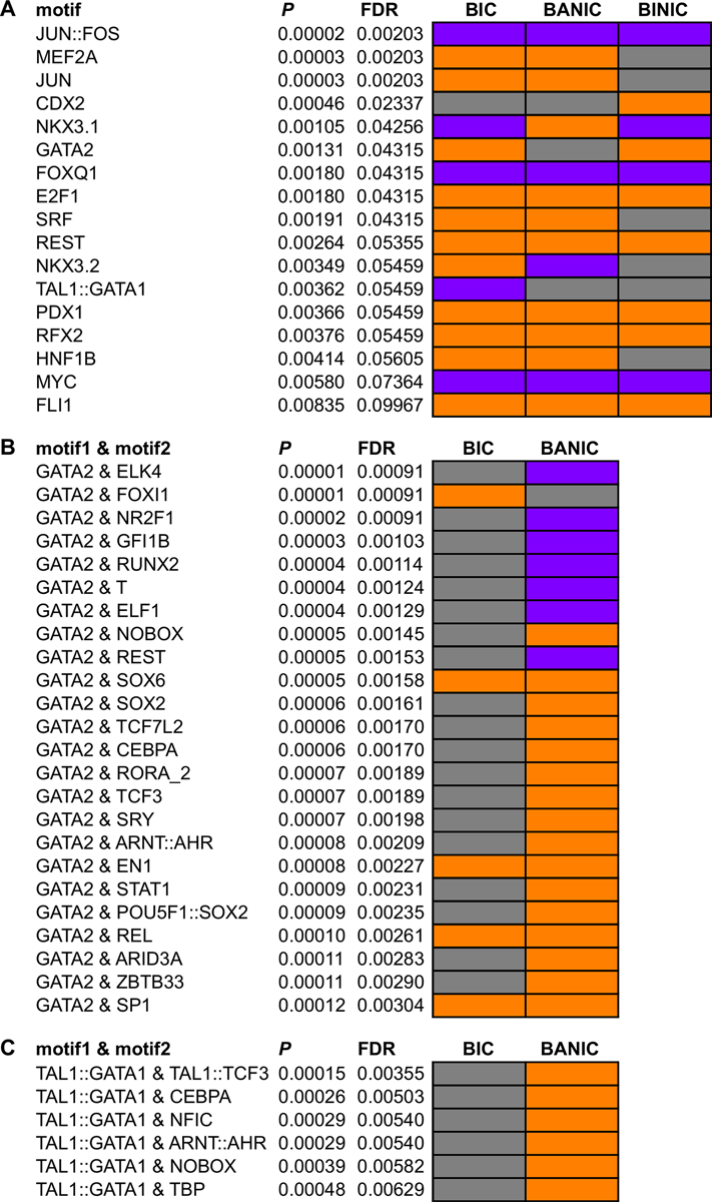
Transcription factor motifs enriched in differentially expressed genes for behavioral groups. (A) Single motifs enriched in at least one of the behavioral groups (BIC, bystanders to interacting conspecifics; BANIC, bystanders attentive to non-interacting conspecifics; or BINIC, bystanders inattentive to non-interacting conspecifics). (B) Pairs of motifs involving GATA2 enriched in BIC and/or BANIC. (C) Pairs of motifs involving TAL1::GATA1 enriched in BIC and/or BANIC. Associations found in each behavior group can be strongest with genes up-regulated (orange) or down-regulated (purple). Grey cells indicate no significance of associations to any group of differentially expressed genes. Significance was calculated using uncorrected (*P*) and corrected (FDR) *P*-values.

Seventeen TF motifs were enriched in at least one of the behavior groups (Fig 3A; see Table 1 for a summary of their functions and respective references). Focusing on the dissimilarities between bystanders to interacting conspecifics and bystanders attentive to non-interacting conspecifics, we observed that only two of the TFs (NKX3.1, NKX3.2) were associated to DE genes in different directions (up- or down-regulated). GATA2 and TAL1::GATA1 were not associated to either up- or down-regulated genes in bystanders attentive to non-interacting conspecifics. However, when considering associations between pairs of motifs (Fig 3B and 3C) we observed that these two TFs were also associated to genes DE in different directions, when comparing bystanders to interacting conspecifics and bystanders attentive to non-interacting conspecifics. These results were not entirely surprising since NKX3 proteins are known to act both as activators and repressors. Results on associations between pairs of TF motifs involving NKX3 proteins also supported this double role in transcription since they showed associations with either up- or down-regulated genes depending on the TF they paired with (S2 Fig). Interestingly, all of the 17 TFs have been associated to neuronal functions or at least shown to be expressed in the brain. Among these, some have an important role in neuronal proliferation, development and/or differentiation (E2F1, GATA2, JUN, JUN::FOS, MEF2A, MYC, REST, SRF), development of nervous system (CDX2, HNF1B, PDX1) and development of sensory organs (FOXQ1, RFX2).

**Fig 4:**
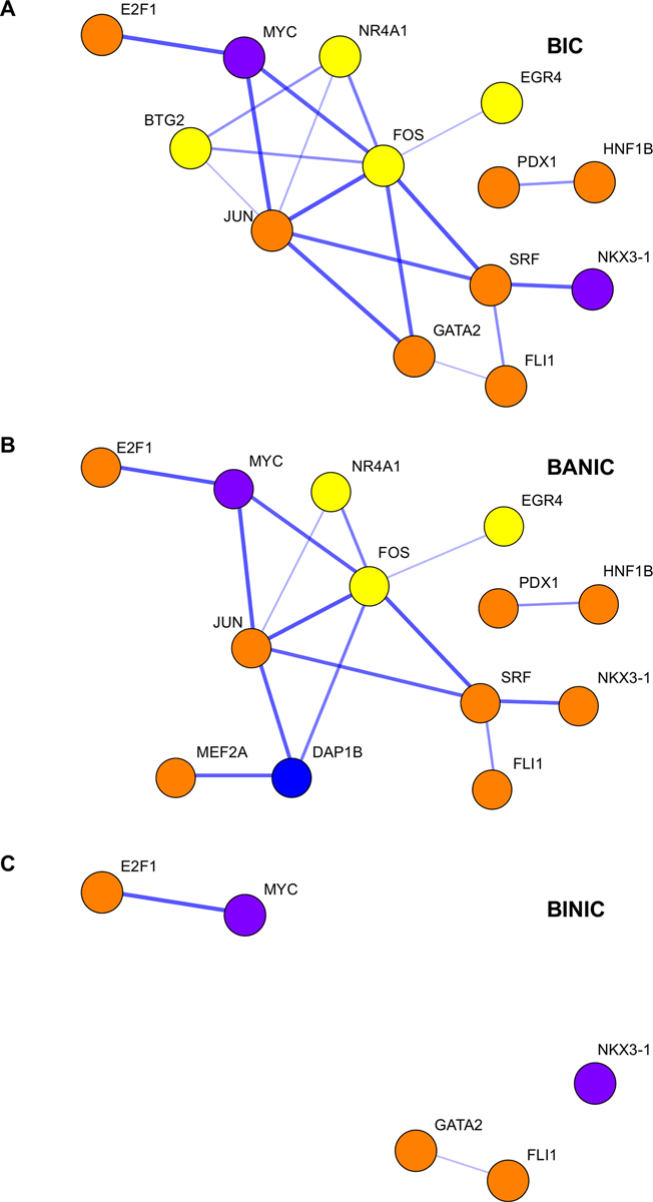
Transcription networks of the different behavioral groups. Networks consisting of DE genes and enriched transcription factors TF for the behavioral groups: (A) bystanders to interacting conspecifics (BIC); (B) bystanders attentive to non-interacting conspecifics (BANIC); and (C) bystanders inattentive to non-interacting conspecifics (BINIC). The thickness of the edges correspond to the confidence score of the genes’ association, yellow nodes indicate up-regulated DE genes, blue nodes indicate down-regulated DE genes, orange nodes indicates TF motifs mainly associated with up-regulated DE genes, and purple indicates TF motifs mainly associated with down-regulated DE genes.

Protein networks were constructed using STRING. This software uses data mining to establish connections between proteins. As such, the establishment of these connections is directly related to information availability. Lack of connections between nodes can result from research biases towards more relevant pathways or any other factor that constrains data collection. Thus, the interpretation of the results should be taken with caution. The networks of bystanders to interacting conspecifics and bystanders attentive to non-interacting conspecifics built using DE genes and enriched TFs (Fig 4A and 4B) had the same number of nodes, but the one of bystanders to interacting conspecifics was composed by more edges (13 nodes and 18 edges vs. 13 nodes and 15 edges), hence having higher density (0.177 vs. 0.160) and lower average path than the one of bystanders attentive to non-interacting conspecifics (2.000 vs. 2.200). The network of bystanders inattentive to non-interacting conspecifics was composed only by 5 nodes and 2 edges (Fig 4C) and was excluded from the remaining network analyses. Networks of bystanders to interacting conspecifics and bystanders attentive to non-interacting conspecifics had very similar topologies (structural correlation coefficient = 1.000). Reassuringly, in both networks the DE up-regulated genes interacted mostly with each other and with TFs enriched in them, whereas DE down-regulated genes seemed to be positioned in proximity with each other and with TFs enriched in them (network assortativity of 0.273 for bystanders to interacting conspecifics and 0.167 for bystanders attentive to non-interacting conspecifics). In both networks, the gene *fos* seemed to have a central position with many connections to various genes (eigenvector centrality of 0.537 and 0.529 for bystanders to interacting conspecifics and bystanders attentive to non-interacting conspecifics, respectively). The gene *jun* also had high values of eigenvector centrality (0.503 for bystanders to interacting conspecifics and 0.517 for bystanders attentive to non-interacting conspecifics).

## Discussion

### Attentional behavioral profiles

Previous work has defined an experimental set up consisting of three treatments: bystander to interacting (fighting) conspecifics; bystander to non-interacting conspecifics; and socially isolated [15]. In the current study, we further defined four different behavioral profiles within these treatments by dividing the bystanders to non-interacting conspecifics into attentive and inattentive profiles. This division was based on a PAM clustering method applied to behavior parameters that have shown significant differences between bystanders and socially isolated individuals (Fig 1). The comparison of the transcriptomes of these four behavioral phenotypes will potentially allow the identification of genetic mechanisms associated with attention processes in general, as indicated by gene expression similarities between bystanders to interacting conspecifics and bystanders attentive to non-intreaction, and with social eavesdropping in particular, as indicated by gene expression patterns exclusive to bystanders to interacting conspecifics.

### Brain transcriptome characterization of the behavioral profiles

Transcriptomic analysis on sampled individuals from the four behavioral groups revealed differences between the socially isolated group and the remaining ones. In particular, gene expression analyses showed that bystanders to interacting conspecifics, bystanders attentive to non-interacting conspecifics and bystanders inattentive to non-interacting conspecifics had 8, 11 and 3 DE genes relative to isolated fish, respectively (Fig 2). Gene set enrichment analyses, using whole genome expression data, also showed the existence of gene sets significantly DE in all the groups. These results indicate that all behavior profiles, even when bystanders did not show attentiveness towards the stimulus, led to transcriptomic responses in the brain that differed from the isolated individuals, albeit in different ways. These differences in gene expression are not explained by putative differences in arousal across treatments, since a previous study, using the same behavioral paradigm, has shown that attentive animals were not more aroused than non-attentive ones, as seen by similar total distance travelled, speed, and cortisol levels between them [15].

Interestingly, four genes (*egr4*, *fos*, *npas4a*, *nr4a1*) that were found to be over-expressed in attentive fish in this study (i.e. bystanders to interacting conspecifics and bystanders attentive to non-interacting conspecifics) have also been found to be differentially expressed in another study in which the brain transcriptome of fish participating in hierarchy-defining fights was examined (Oliveira et al, 2015). In this other study, we found one order of magnitude more DE genes than in the current experiment (168 vs. 16). This was expected since interacting with conspecifics should lead to more neurogenomic changes than just observing conspecifics. These four genes are all neural activity-dependent immediate early genes, which could indicate that their activation merely reflects task-related brain activity. However, they are also known to have a role in neuronal plasticity [41] and contextual and fear memory formation [42,45,57,58], hence suggesting that neurogenomic changes observed in attentive bystanders are part of the changes observed in individuals actively participating in a social interaction, and that these changes are likely to be related to acquisition of social information.

The hierarchical clustering of the DE genes of all behavior groups pooled together showed a well-defined group of 8 genes with similar profile expression across the 12 individuals (btg2, dnajb5, egr4, fos, msh4, npas4a, npas4b, nr4a1), suggesting that these genes share a co-expression pattern. Notably, this set of co-expressed genes included all the over-expressed genes in bystanders to interacting conspecifics, suggesting that they all play a relevant role in this behavioral profile. The interaction between four of those genes (*btg2*, *egr4*, *fos*, and *nr4a1*) was confirmed by STRING results (Fig 4A). Moreover, by reducing STRING confidence score to low (> 0.150), we further obtained evidence for interactions including proteins NPAS4 and MSH4 (results not shown). Together these results support the notion of a change in the neurogenomic network after exposure to conspecifics, in which some of the key players are the over-expressed genes of bystanders to interacting conspecifics, including *btg2*, *egr4*, *fos*, *npas4a*, and *nr4a1*, also found to be differentially expressed in individuals involved in fighting interactions (Oliveira et al, 2015).

### Brain transcriptome comparison between Bystanders to interacting conspecifics and Bystanders attentive to non-interacting conspecifics

Results from the behavior experiment and the analyses of DE genes suggested that inattentive individuals had a behavior profile and a neurogenomic state closer to the socially isolated individuals, whereas the two groups of attentive individuals had both similar behavior profiles and similar neurogenomic states. Nevertheless, we found important differences between bystanders to interacting conspecifics and bystanders attentive to non-interacting conspecifics, which may be associated with the acquisition of eavesdropped information by the former. In this sense the 4 over-expressed genes found uniquely in bystanders to interacting conspecifics can be considered to be specifically associated with social eavesdropping. From these, *btg2* and *npas4b* have been shown to have a role in neuronal plasticity and contextual and fear memory formation [40,45,57], *msh4* has been associated to nervous system tumors [59] and *dnajb5* has been linked to cellular stress regulation [60] and to the circadian neuronal circuit in *Drosophila* [32]. Interestingly, GAGE analyses has also shown a differential expression in bystanders to interacting conspecifics, but not in bystanders attentive to non-interacting conspecifics, in both GO term “response to stress” and Wikipathway term “exercise-induced circadian regulation” (S10–S15 Tables). The other gene set that was differentially expressed uniquely in bystanders to interacting conspecifics was GO “adrenergic receptor activity”, which is related to the “fight-or-flight” response.

The analyses of transcription factors, using a more relaxed definition of DE genes, showed again strong similarities between bystanders to interacting conspecifics and bystanders attentive to non-interacting conspecifics (Fig 3). However, this analysis also presented important differences between the two behavior groups: four transcription factor motifs from proteins NKX3.1, NKX3.2, GATA2 and complex TAL1::GATA1 were over-represented in genes differentially expressed in opposite directions in bystanders to interacting conspecifics and bystanders attentive to non-interacting conspecifics. These results were not unexpected since NKX3 proteins can act either as repressor or activator [61–63] and have been shown to be expressed in the brain [64]. GATA2 and TAL1 have also been shown to be expressed in the brain and they have particularly important roles in neuronal differentiation [36,39]. Finally, the network analyses have shown that although the networks of both groups are similar (structural correlation coefficient close to 1) with proteins FOS and JUN being important players, the one of bystanders to interacting conspecifics is composed by more edges and have higher density (Fig 4). Together these results suggest that the neurogenomic responses in social eavesdropping (bystanders to interacting conspecifics) and in bystanders to non-interacting conspecifics share considerable similarities, which reflect attention processes, but also that on the whole both groups have distinct neurogenomic profiles, which can be related to the eavesdropping of social information. Some areas that may explain the mechanisms behind these differences are suggested. Pathways related to stress and flight-or-fight response seem to be obvious candidates, but more surprisingly the circadian neuronal circuit may also have a role. Epigenetic mechanisms provided by transcription factors that function both as repressors and activators, for example the NKX3 proteins, seem to be important as well.

### Brain transcriptome of Bystanders inattentive to non-interacting conspecifics

Regarding the group of bystanders inattentive to non-interacting conspecifics, although their behavioral profile seems to be very close to isolated fish (Fig 1), extensive transcriptomic analyses revealed important differences. This group had only 3 DE genes (all over-expressed) in relation to isolated fish (Fig 2), however, these genes have known important neuronal functions. Protocadherin alfa genes *pcdh2ab7* and *pcdhga10* have a role in self-recognition by individual neurons [30], being important in establishing neuronal connections in the brain [65], and *osbpl1a* has been shown to be expressed at considerable high levels in cortical areas of the human brain [31] and to regulate cellular cholesterol metabolism in vitro [66]. Moreover, GAGE analyses showed DE gene sets in areas similar to the remaining groups, namely, cholesterol biosynthesis, metabolism, transcription and sensory organs (S10–S15 Tables). These results suggest that the presence of conspecifics affects bystanders irrespective of them being attentive or not. Although the transcriptomic brain activity of this group seemed to be unrelated to neuronal plasticity, the presence of a conspecific still triggers important responses.

## Conclusions

Overall, the results presented here allow us to identify three types of transcriptomic responses associated with the experimentally defined behavioural profiles. First, there are transcriptomic changes that are common to all the behavior groups (i.e. bystanders to interacting conspecifics, bystanders attentive to non-interacting conspecifics, bystanders inattentive to non-interacting conspecifics), which may indicate that they are linked to a bystander response in the presence of conspecifics, irrespective of attentiveness. Second, there is a set genes that is responding on both attentive groups, which includes genes related to neuronal plasticity and memory (e.g. *fos*, *jun*, *npas4a*, *nr4a1*, *egr4*). This result suggests that attentive individuals are forming social memories in response to the acquisition of information from conspecifics. Third, a set of genes (e.g. *btg2*, *npas4b*, *dnajb5* and *msh4*) was uniquely associated to bystanders to interacting conspecifics, which may indicate that these genes are particularly important in social eavesdropping of fighting interactions. Thus, social information seems to trigger specific genomic responses depending on its content conveying static cues from non-interacting conspecifics or dynamic cues from interacting conspecifics.

Finally, we would like to finish with two cautionary notes. First, it should be noted that the observed transcriptome responses in this study relied only on visual information. However, chemical information is also used by zebrafish in the social domain. For example, zebrafish can discriminate between familiar and unfamiliar individuals based on odor cues only [67]. Thus, not all social cues were accessible to bystanders. Nevertheless, visual cues alone were sufficient to convey relevant social information that drives attention and to trigger a transcriptomic response. Future studies can further dissect the relative contributions of different sensory modalities for social information use. Secondly, the use of whole brain samples in this study should be seen as a first approach to the study of the genomics of social information use in zebrafish. Attention is a cognitive process known to recruit neural networks that span across multiple brain structures (e.g. telencephalon, optic tectum) both in mammals and in fish [68,69]. Therefore, by characterizing the whole-brain transcriptome of animals attentive to social stimuli we are focusing more on the overall pattern of gene expression emerging from such networks, rather then on regional changes within the network. Now that key genes have been identified at the whole-network level future studies can use this information to further dissect candidate brain regions of social attention networks in fish.

## Supporting Information

S1 Fig Spatial and directional patterns of the focal fish selected for transcriptomic analysis. Linear histograms plus 2D heatmaps of time spent in each position of the arena (left), polar directional histograms (right). Groups considered: bystanders to interacting conspecifics (BIC, magenta); bystanders attentive to non-interacting conspecifics (BANIC, green); bystanders inattentive to non-interacting conspecifics (BINIC, lime); and isolated fish (ISOL, blue). Heatmaps are scaled from maximum relative value (red) to minimum relative value (dark blue). Linear and polar histograms represented in absolute arbitrary scale.
(TIF)

S2 Fig Transcription factor motif pairs found in significant meta-associations. Pairs of motifs involving transcription factors NKX3.1 (A); and KX3.2 (B). The strongest association found in the considered behavior group (bystanders to interacting conspecifics, BIC; and bystanders attentive to non-interacting conspecifics, BANIC) can be with up-regulated (orange) or down-regulated (purple). Grey cells indicate no significance of associations to any group of DE genes. Significance was calculated using uncorrected (*P*) and corrected (FDR) *P*-values.
(TIF)

S1 Methods (DOC)

S1 Table Genes differentially expressed in the brain of zebrafish in response to eavesdropping interacting conspecifics [FC > log_2_(1.1) and FDR < 0.05]. The gene list is sorted by FDR.
(DOC)

S2 Table Genes differentially expressed in the brain of attentive zebrafish in response to observing non-interacting conspecifics [FC > log_2_(1.1) and FDR < 0.05]. The gene list is sorted by FDR. (DOC)

S3 Table Genes differentially expressed in the brain of inattentive zebrafish in response to observing non-interacting conspecifics [FC > log_2_(1.1) and FDR < 0.05]. The gene list is sorted by FDR.
(DOC)

S4 Table KEGG gene sets over-represented in the differentially expressed genes [*P*-value < 0.1] for bystanders to interacting conspecifics (BIC), bystanders attentive to non-interacting conspecifics (BANIC) and bystanders inattentive to non-interacting conspecifics (BINIC). BINIC had no gene set over-represented**. Gene sets list sorted by *P*-value.**
(DOC)

S5 Table Wikipathway gene sets over-represented in the differentially expressed genes [*P*-value < 0.1] for bystanders to interacting conspecifics (BIC), bystanders attentive to non-interacting conspecifics (BANIC) and bystanders inattentive to non-interacting conspecifics (BINIC). BINIC had no gene set over-represented**. Gene sets list sorted by *P*-value.**
(DOC)

S6 Table GO Biological process gene sets over-represented in the differentially expressed genes [*P*-value < 0.1] for bystanders to interacting conspecifics (BIC), bystanders attentive to non-interacting conspecifics (BANIC) and bystanders inattentive to non-interacting conspecifics (BINIC). Gene sets list sorted by *P*-value.
(DOC)

S7 Table GO Cellular component gene sets over-represented in the differentially expressed genes [*P*-value < 0.1] for bystanders to interacting conspecifics (BIC), bystanders attentive to non-interacting conspecifics (BANIC) and bystanders inattentive to non-interacting conspecifics (BINIC). Gene sets list sorted by *P*-value.
(DOC)

S8 Table GO Molecular function gene sets over-represented in the differentially expressed genes [*P*-value < 0.1] for bystanders to interacting conspecifics (BIC), bystanders attentive to non-interacting conspecifics (BANIC) and bystanders inattentive to non-interacting conspecifics (BINIC). Gene sets list sorted by *P*-value.
(DOC)

S9 Table Chromosome location gene sets over-represented in the differentially expressed genes [*P*-value < 0.1] for bystanders to interacting conspecifics (BIC), bystanders attentive to non-interacting conspecifics (BANIC) and bystanders inattentive to non-interacting conspecifics (BINIC). Gene sets list sorted by *P*-value.
(DOC)

S10 Table KEGG gene sets differentially expressed considering under- and over-expressed genes [*P*-value < 0.1] for bystanders to interacting conspecifics (BIC), bystanders attentive to non-interacting conspecifics (BANIC) and bystanders inattentive to non-interacting conspecifics (BINIC). Gene sets list sorted by *P*-value.
(DOC)

S11 Table Wikipathway gene sets differentially expressed considering under- and over-expressed genes [*P*-value < 0.1] for bystanders to interacting conspecifics (BIC), bystanders attentive to non-interacting conspecifics (BANIC) and bystanders inattentive to non-interacting conspecifics (BINIC). Gene sets list sorted by *P*-value.
(DOC)

S12 Table GO Biological process gene sets differentially expressed considering only over-expressed genes [*P*-value < 0.1] for bystanders to interacting conspecifics (BIC), bystanders attentive to non-interacting conspecifics (BANIC) and bystanders inattentive to non-interacting conspecifics (BINIC). Gene sets list sorted by *P*-value.
(DOC)

S13 Table GO Cellular Compartment process gene sets differentially expressed considering only over-expressed genes [*P*-value < 0.1] for bystanders to interacting conspecifics (BIC), bystanders attentive to non-interacting conspecifics (BANIC) and bystanders inattentive to non-interacting conspecifics (BINIC). Gene sets list sorted by *P*-value.
(DOC)

S14 Table GO Molecular Function process gene sets differentially expressed considering only over-expressed genes [*P*-value < 0.1] for bystanders to interacting conspecifics (BIC), bystanders attentive to non-interacting conspecifics (BANIC) and bystanders inattentive to non-interacting conspecifics (BINIC). Gene sets list sorted by *P*-value.
(DOC)

S15 Table Chromosome location gene sets differentially expressed considering under- and over-expressed genes (*P*-value < 0.1) for bystanders to interacting conspecifics (BIC), bystanders attentive to non-interacting conspecifics (BANIC) and bystanders inattentive to non-interacting conspecifics (BINIC). Gene sets list sorted by *P*-value.
(DOC)
