# Supplementary figures and images for: Brain Transcriptomic Response to Social Eavesdropping in Zebrafish (Danio rerio)
Source: PLoS One. 2015 Dec 29;10(12):e0145801. doi: 10.1371/journal.pone.0145801 (PMC4700982; doi:10.1371/journal.pone.0145801)

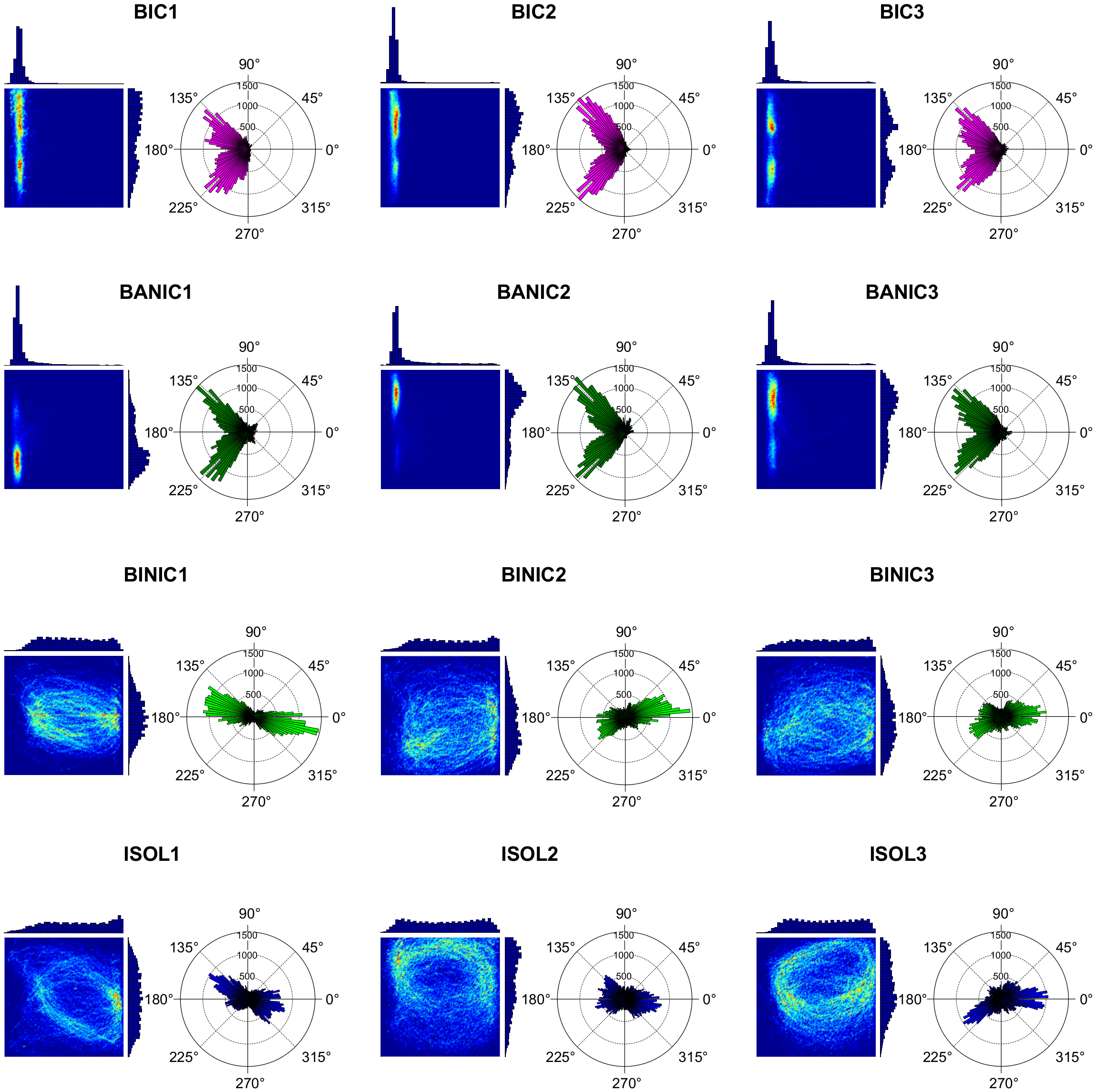

Supplement: S1 Fig — Linear histograms plus 2D heatmaps of time spent in each position of the arena (left), polar directional histograms (right). Groups considered: bystanders to interacting conspecifics (BIC, magenta); bystanders attentive to non-interacting conspecifics (BANIC, green); bystanders inattentive to non-interacting conspecifics (BINIC, lime); and isolated fish (ISOL, blue). Heatmaps are scaled from maximum relative value (red) to minimum relative value (dark blue). Linear and polar histograms represented in absolute arbitrary scale. (TIF) [file pone.0145801.s001.tif]

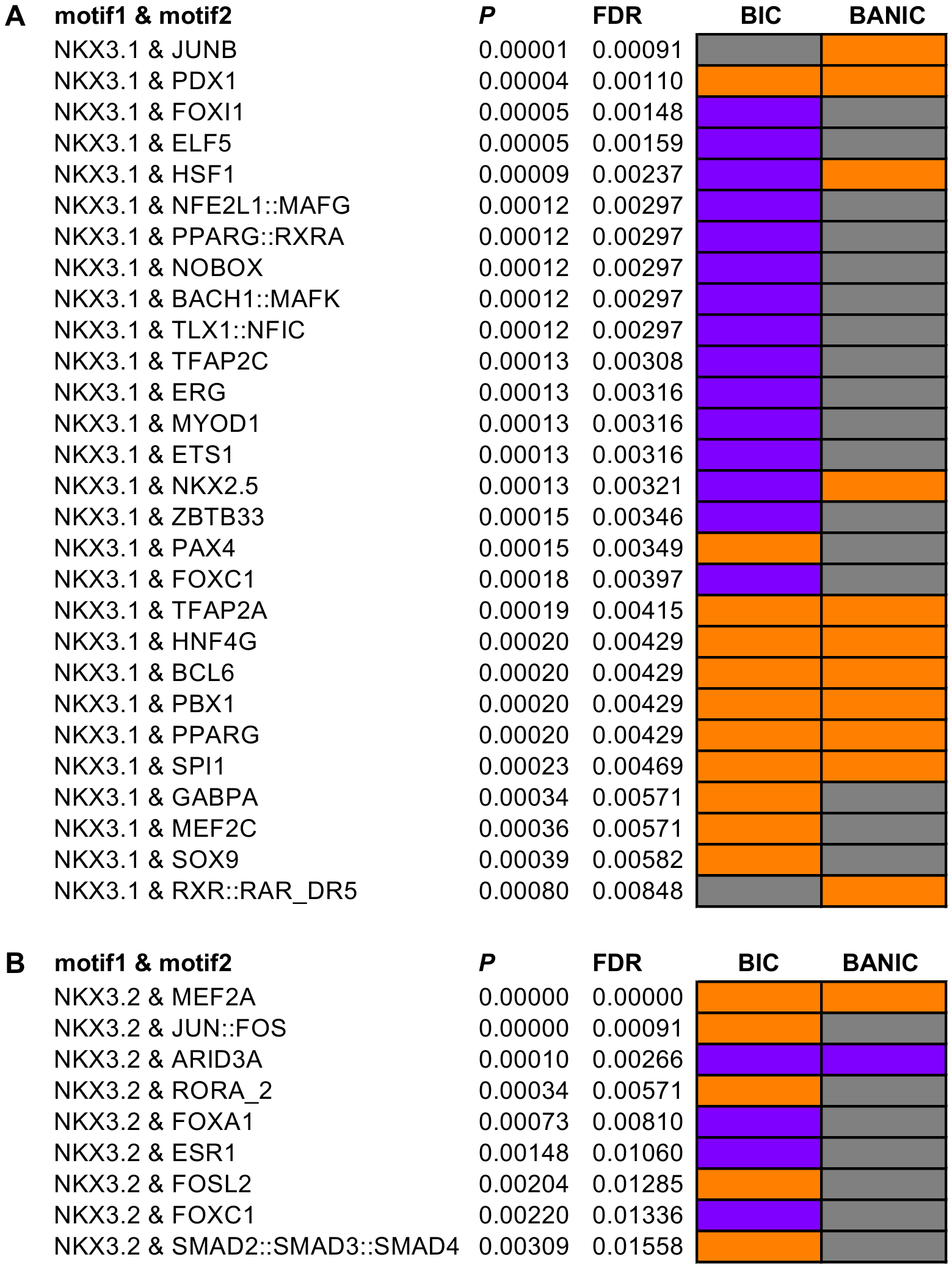

Supplement: S2 Fig — Pairs of motifs involving transcription factors NKX3.1 (A); and KX3.2 (B). The strongest association found in the considered behavior group (bystanders to interacting conspecifics, BIC; and bystanders attentive to non-interacting conspecifics, BANIC) can be with up-regulated (orange) or down-regulated (purple). Grey cells indicate no significance of associations to any group of DE genes. Significance was calculated using uncorrected (P) and corrected (FDR) P-values. (TIF) [file pone.0145801.s002.tif]
