# Supplementary material for: Brain Transcriptomic Response to Social Eavesdropping in Zebrafish (Danio rerio)
Source: PLoS One. 2015 Dec 29;10(12):e0145801. doi: 10.1371/journal.pone.0145801 (PMC4700982; doi:10.1371/journal.pone.0145801)
Supplement: S1 Methods — (DOC) [file pone.0145801.s003.doc]

**S1 Methods**

***Brain transcriptomic response to social eavesdropping in zebrafish***

*João Sollari Lopes, Rodrigo Abril-de-Abreu, Rui F. Oliveira*

**Microarray annotation.** Affymetrix zebrafish gene 1.1 ST array strip are constituted by probes distributed across the full length of the genome, providing a complete and accurate estimate of overall gene expression. They contain 300 000 25-mer oligonucleotide probes corresponding to 75 196 probe sets. Using Affymetrix NetAffx annotation file v33.3 ([http://www.affymetrix.com](http://www.affymetrix.com/)) and Bioconductor datasets, we obtained 23 453 probe sets that identify one and only one Entrez Gene ID (corresponding to 21 485 unique Entrez IDs). Using NCBI Gene database ([http://www.ncbi.gov](http://www.ncbi.gov/)), we filtered-out discontinued Entrez IDs and replaced the deprecated ones, obtaining 23 292 probe sets and 21 224 unique Entrez IDs. These probe sets were used to select the differentially expressed (DE) genes. Using both NCBI Gene database and biomart ([www.biomart.org](http://www.biomart.org/)), we identified a total of 20 944 unique Entrez IDs with one and only one chromosome location. These genes were used for the promoter region analysis. Annotation was performed using Bioconductor (1) packages “biomaRt” (2) and “reutils”.

**Microarray data analysis.** In order to study changes in gene expression in the four representative behavior groups (bystanders to interacting conspecifics, BIC; bystanders attentive to non-interacting conspecifics, BANIC; bystanders inattentive to non-interacting conspecifics, BINIC; and isolated fish, ISOL), twelve Affymetrix zebrafish gene 1.1 ST array strip microarrays (three samples per group) were used. Quality control of the raw data was performed by: visualizing the mapped probe intensity images; smoothed histogram and box-plots of the intensities; assessing intensity bias from plotting average intensity against base positions and GC-contents and from MAplots (3); and by looking at the residuals and weights associated to the probes after fitting a probe-level model (4). These tests were performed using R (5) and the Bioconductor (1) package “oligo” (6).

The signal intensity of the microarrays was normalized using the standard Robust-Multichip average normalization, considering background subtraction, quantile normalization and median-polish summarization. Following standard procedures (7), the normalized data was filtered using nonspecific filtering by choosing only the transcripts annotated with corresponding Entrez Gene IDs, by removing duplicate Entrez Gene IDs with lower gene expression, and by considering only the transcripts with gene expressions that possessed a standard deviation, across the twelve samples, higher than the median standard deviation of all considered genes. Thus, from 23 292 probe sets, 10 748 transcripts were used, each corresponding to one and only one gene.

The selection of differentially expressed (DE) genes was performed considering an experimental design with the group ISOL as a reference and using separately the remaining groups (BIC, BANIC and BINIC). A moderate test statistic implemented in the Bioconductor package “limma” (8) was used, which consisted of fitting a linear model for every gene and performing an empirical Bayes moderation of the standard errors. The *P*-values were adjusted using Benjamini and Hochberg's false discovery rate (FDR, Benjamini & Hochberg 1995). The threshold for DE genes was set to FDR < 0.05 and fold-change ≥ 2 or ≤ 0.5 (log_2_ fold-change ≥ 1 or ≤ -1). Considering the expression levels of the pooled group of DE genes for the behavior groups (BIC, BANIC and BINIC), a hierarchical clusters of both samples and genes were created. Following previous studies (10), the clustering was performed using average linkage and Manhattan distances on normalized values (i.e. values were subtracted by the variable's mean value and divided by the variable's standard deviation).

For each behavior group, we performed hypergeometric tests to assess if their DE genes were over-represented in some pathways or functional categories. These analyses require the definition of a universe of possible genes, and the set of genes that resulted from the nonspecific filtering was considered. The set of genes considered came from various collections, namely, KEGG (11), Wikipathway (12), GO (13) and chromosome locations. For the analyses using collections from GO and KEGG and the chromosome locations, the Bioconductor databases and packages “GOstats” and “Category” (14) were used. For Wikipathways, the same procedure was followed, except that the collections were created using core packages from R. The threshold for overrepresentation gene sets was *P*-value < 0.1. For the analyses of GO collections, the results were further filtered by removing parent categories with significant (*P*-value < 0.1) children categories constituted by the same DE genes.

We also performed Generally Applicable Gene-set analysis (GAGE, Luo et al. 2009) for each behavior group. This approach considers the whole universe of possible genes (i.e. the 10 748 genes resulting from the nonspecific filtering) and uses a two-sample t-test to account for both gene set variance and background variance. Furthermore, GAGE uses genes as the sampling unit (competitive method), allowing for the use of small sample sizes per group. As done for ORA, gene sets came from GO, KEGG, WikiPathways and chromosome locations. GAGE also allows for choosing between single (i.e. consider only up-regulated genes) and both direction (i.e. consider up- and down-regulated genes) analysis. Single analysis are recommended for experimental sets (e.g. GO), whereas both direction analysis are recommended for canonical pathways (e.g. KEGG, Wikipathay) and chromosome locations. Comparisons between behavior groups and reference ISOL was performed considering the average of the replicates, and gene sets that were too small (< 10 members) or too big (> 500 members) were discarded. The threshold for DE gene sets was *P*-value < 0.1. One potential problem in assessing DE gene sets is the overlap of genes between gene sets, which can lead to false identification of overlapping gene sets as significantly DE. In order to circumvent this problem and diagnose the effect of overlapping, we used function “esset.grp” from Bioconductor package “gage”. This method considers the overlap between core genes (i.e. member genes that highly contributed to the significance of the gene set) to calculate a *P*-value based on network graphs. As recommended by the authors, we set the *P*-value threshold to 10^-10^. These analysis were performed using Bioconductor packages “GSEABase” and “gage”.

**Promoter region analysis and transcription network.** The algorithm used to find transcription factor binding sites enriched in the DE genes of the behavior groups was as follow:

1. Download whole genome of zebrafish from UCSC (http://genome.ucsc.edu) with repeats masked using RepeatMasker and Tandem Repeats Finder with a period of 12 or less. Consider only chromosomes 1 to 25;

2. Download motif collection Jaspar Vertebrate Core from Jaspar (16), which consists of 205 non-redundant motifs;

3. Calculate motif scores using Stubb (17) for every motif of the motif collection in every 500bp window of the genome with a shift of 250bp;

4. Assign the windows to one of 20 bins according to their GC content. For each motif, rank normalize windows according to their motif score (*P*-value = 1 – rank/N, where N is the number of windows in the bin);

5. For each annotated gene, consider an upstream region of 5 000bp and find, for each motif, the window inside the region with most significant *P*-value. For each motif, define motif target genes as the genes with the most significant *P*-value < 0.0025;

6. For each motif and pair of motifs, calculate the enrichment of moderated up- and down-regulated genes (*P*-value < 0.01) for each behavior group (BIC, BANIC and BINIC), while considering the interactions between pairs of motifs implemented by *cis*-Metalysis (18). Calculate meta-associations between the best associations found in each social context using the “flexible” mode in *cis*-Metalysis. Correct p-values for multiple comparisons using FDR;

7. Run STRING 9.1 (19) on the list of differentially expressed genes (FDR < 0.05) and enriched transcription factors (FDR < 0.1) for all behavior groups (medium confidence).

The chosen significance thresholds varied along the analysis. This was done to respect authors’ recommendations. In order to define genes strongly DE we assumed the standard FDR < 0.05. The list of moderate DE to be used in cis-Metalysis was defined using the recommended *P*-value < 0.01 (18). The threshold to define the motif target genes considered was such that these lists were composed by about 500 genes (P-value < 0.0025, Whitney et al. 2014). Finally, the threshold for defining enriched transcription factors was chosen to be a relaxed FDR < 0.1, so that to favor the control of false negatives, instead of false positives [note, however, that this threshold is still more conservative than the one proposed by the authors (18)].

**Bibliography**

1. Gentleman RC, Carey VJ, Bates DM, Bolstad B, Dettling M, Dudoit S, et al. Bioconductor: open software development for computational biology and bioinformatics. Genome Biol. 2004;5(10):R80.

2. Durinck S, Spellman PT, Birney E, Huber W. Mapping identifiers for the integration of genomic datasets with the R/Bioconductor package biomaRt. Nat Protoc. 2009;4(8):1184–91.

3. Dudoit S, Yang YH, Callow MJ, Speed TP. Statistical methods for identifying differentially expressed genes in replicated cDNA microarray experiments. Stat Sin. 2002;12:111–39.

4. Bolstad B. Probe-level model based test statistics for detecting differential expression. University of California, Berkeley; 2004.

5. R Development Core Team. R: A language and environment for statistical computing. [Internet]. R Foundation for Statistical Computing, Vienna, Austria. 2013. Available from: http://www.r-project.org/

6. Carvalho BS, Irizarry RA. A framework for oligonucleotide microarray preprocessing. Bioinformatics. 2010;26(19):2363–7.

7. Bourgon R, Gentleman R, Huber W. Independent filtering increases detection power for high-throughput experiments. Proc Natl Acad Sci U S A. 2010;107(21):9546–51.

8. Smyth G. limma: Linear Models for Microarray Data. In: Gentleman R, Carey V, Huber W, Irizarry R, Dudoit S, editors. Bioinformatics and Computational Biology Solutions Using R and Bioconductor. Springer-Verlag; 2005. p. 397–420.

9. Benjamini Y, Hochberg Y. Controlling the False Discovery Rate: A Practical and Powerful Approach to Multiple Testing. J R Stat Soc Ser B. 1995;57(1):289–300.

10. Datta S, Datta S. Comparisons and validation of statistical clustering techniques for microarray gene expression data. Bioinformatics. 2003;19(4):459–66.

11. Kanehisa M, Goto S, Sato Y, Kawashima M, Furumichi M, Tanabe M. Data, information, knowledge and principle: Back to metabolism in KEGG. Nucleic Acids Res. 2014;42(D1).

12. Kelder T, Pico AR, Hanspers K, Van Iersel MP, Evelo C, Conklin BR. Mining biological pathways using WikiPathways web services. PLoS One. 2009;4(7).

13. Ashburner M, Ball CA, Blake JA, Botstein D, Butler H, Cherry JM, et al. Gene ontology: tool for the unification of biology. The Gene Ontology Consortium. Nat Genet. 2000;25(1):25–9.

14. Falcon S, Gentleman R. Using GOstats to test gene lists for GO term association. Bioinformatics. 2007;23(2):257–8.

15. Luo W, Friedman MS, Shedden K, Hankenson KD, Woolf PJ. GAGE: generally applicable gene set enrichment for pathway analysis. BMC Bioinformatics. 2009;10:161.

16. Mathelier A, Zhao X, Zhang AW, Parcy F, Worsley-Hunt R, Arenillas DJ, et al. JASPAR 2014: An extensively expanded and updated open-access database of transcription factor binding profiles. Nucleic Acids Res. 2014;42(D1).

17. Sinha S, van Nimwegen E, Siggia ED. A probabilistic method to detect regulatory modules. Bioinformatics. 2003;19(Suppl 1):i292–301.

18. Ament SA, Blatti CA, Alaux C, Wheeler MM, Toth AL, Le Conte Y, et al. New meta-analysis tools reveal common transcriptional regulatory basis for multiple determinants of behavior. Proc Natl Acad Sci. 2012;109(26):E1801–10.

19. Franceschini A, Szklarczyk D, Frankild S, Kuhn M, Simonovic M, Roth A, et al. STRING v9.1: Protein-protein interaction networks, with increased coverage and integration. Nucleic Acids Res. 2013;41(D1):D808–15.

20. Whitney O, Pfenning AR, Howard JT, Blatti CA, Liu F, Ward JM, et al. Core and region-enriched networks of behaviorally regulated genes and the singing genome. Science. 2014;346(6215):1256780.
