# Supplementary material for: Brain Transcriptomic Response to Social Eavesdropping in Zebrafish (Danio rerio)
Source: PLoS One. 2015 Dec 29;10(12):e0145801. doi: 10.1371/journal.pone.0145801 (PMC4700982; doi:10.1371/journal.pone.0145801)
Supplement: S1 Table — The gene list is sorted by FDR. (DOC) [file pone.0145801.s004.doc]

**S1 Table**. Genes differentially expressed in the brain of zebrafish in response to eavesdropping interacting conspecifics [FC > log_2_(1.1) and FDR < 0.05]. The gene list is sorted by FDR.

| Name | FC^a^ | FDR | Entrez ID | Gene Symbol | Description |
| --- | --- | --- | --- | --- | --- |
| 13015447 | 2.24 | 0.001 | 724016 | npas4a | neuronal PAS domain protein 4a |
| 13047782 | 1.52 | 0.002 | 559917 | msh4 | mutS homolog 4 (E. coli) |
| 13143256 | 1.57 | 0.003 | 795099 | EGR4 (2 of 2)^a^ | early growth response 4 |
| 13105945 | 1.96 | 0.003 | 394198 | fos | v-fos FBJ murine osteosarcoma viral oncogene homolog |
| 13110394 | 1.41 | 0.003 | 100534657 | npas4b | neuronal PAS domain protein 4 |
| 13141648 | 1.60 | 0.010 | 431720 | nr4a1 | nuclear receptor subfamily 4, group A, member 1 |
| 13124986 | 1.28 | 0.013 | 30079 | btg2 | B-cell translocation gene 2 |
| 13107726 | 1.78 | 0.016 | 641576 | DNAJB5 (2 of 2)^a^ | DnaJ (Hsp40) homolog, subfamily B, member 5-like |
| FC, fold change; FDR, false discovery change  ^a^ - log_2_ fold-change, negative is under-expressed, positive is over-expressed.  ^b^ - gene symbol from Ensembl | | | | | |
