# Supplementary material for: Brain Transcriptomic Response to Social Eavesdropping in Zebrafish (Danio rerio)
Source: PLoS One. 2015 Dec 29;10(12):e0145801. doi: 10.1371/journal.pone.0145801 (PMC4700982; doi:10.1371/journal.pone.0145801)
Supplement: S2 Table — (DOC) [file pone.0145801.s005.doc]

**S2 Table**. Genes differentially expressed in the brain of attentive zebrafish in response to observing non-interacting conspecifics [FC > log_2_(1.1) and FDR < 0.05]. The gene list is sorted by FDR.

| Name | FC^a^ | FDR | Entrez ID | Gene Symbol | Description |
| --- | --- | --- | --- | --- | --- |
| 13007436 | 2.56 | 0.000 | 493593 | pcdh2ab7 | protocadherin 2 alpha b 7 |
| 12959481 | 2.82 | 0.000 | 572221 | ZNF507 (2 of 5) ^b^ | Zinc finger protein 507 |
| 13172083 | -3.94 | 0.000 | 100137114 | ftr50 | finTRIM family, member 50 |
| 13007420 | 1.61 | 0.001 | 100535907 | pcdhga10 | protocadherin gamma-A10-like |
| 13136272 | -1.60 | 0.004 | 563485 | soga3b | SOGA family member 3b |
| 13143256 | 1.15 | 0.006 | 795099 | EGR4 (2 of 2) ^b^ | early growth response 4 |
| 13015447 | 1.81 | 0.012 | 724016 | npas4a | neuronal PAS domain protein 4a |
| 13162324 | -1.39 | 0.012 | 777611 | C25HXorf38 (1 of 2)^b^ | chromosome X open reading frame 38 |
| 13105945 | 1.57 | 0.012 | 394198 | fos | v-fos FBJ murine osteosarcoma viral oncogene homolog |
| 13141648 | 1.06 | 0.018 | 431720 | nr4a1 | nuclear receptor subfamily 4, group A, member 1 |
| 13263259 | -1.04 | 0.031 | 58094 | dap1b | death associated protein 1b |
| FC, fold change; FDR, false discovery change  ^a^ – log_2_ fold-change, negative is under-expressed, positive is over-expressed.  ^b^ – gene symbol from Ensembl | | | | | |
