# Supplementary material for: Brain Transcriptomic Response to Social Eavesdropping in Zebrafish (Danio rerio)
Source: PLoS One. 2015 Dec 29;10(12):e0145801. doi: 10.1371/journal.pone.0145801 (PMC4700982; doi:10.1371/journal.pone.0145801)
Supplement: S3 Table — The gene list is sorted by FDR. (DOC) [file pone.0145801.s006.doc]

**S3 Table**. Genes differentially expressed in the brain of inattentive zebrafish in response to observing non-interacting conspecifics [FC > log_2_(1.1) and FDR < 0.05]. The gene list is sorted by FDR.

| Name | FC^a^ | FDR | Entrez ID | Gene Symbol | Description |
| --- | --- | --- | --- | --- | --- |
| 13007436 | 2.55 | 0.000 | 493593 | pcdh2ab7 | protocadherin 2 alpha b 7 |
| 13078177 | 2.44 | 0.000 | 100331149 | OSBPL1A (2 of 2)^b^ | oxysterol-binding protein-related protein 1-like |
| 13007420 | 1.38 | 0.019 | 100535907 | pcdhga10 | protocadherin gamma-A10-like |
| FC, fold change; FDR, false discovery change  ^a^ – log_2_ fold-change, negative is under-expressed, positive is over-expressed.  ^b^ – gene symbol from Ensembl | | | | | |
