# Supplementary material for: Brain Transcriptomic Response to Social Eavesdropping in Zebrafish (Danio rerio)
Source: PLoS One. 2015 Dec 29;10(12):e0145801. doi: 10.1371/journal.pone.0145801 (PMC4700982; doi:10.1371/journal.pone.0145801)
Supplement: S4 Table — BINIC had no gene set over-represented. Gene sets list sorted by P-value. (DOC) [file pone.0145801.s007.doc]

**S4 Table.** KEGG gene sets over-represented in the differentially expressed genes [*P*-value < 0.1] for bystanders to interacting conspecifics (BIC), bystanders attentive to non-interacting conspecifics (BANIC) and bystanders inattentive to non-interacting conspecifics (BINIC). BINIC had no gene set over-represented. Gene sets list sorted by *P*-value.

| Group | ID | Description | *P*-value | Counts | Size | Up | Dn |
| --- | --- | --- | --- | --- | --- | --- | --- |
| BIC | 4010 | **MAPK signaling pathway** | 0.00888 | 2 | 107 | 2 | 0 |
|  | 3018 | RNA degradation | 0.03701 | 1 | 24 | 1 | 0 |
|  | 4620 | Toll-like receptor signaling pathway | 0.06417 | 1 | 42 | 1 | 0 |
| BANIC | 4010 | **MAPK signaling pathway** | 0.00307 | 2 | 107 | 2 | 0 |
|  | 4620 | Toll-like receptor signaling pathway | 0.04324 | 1 | 42 | 1 | 0 |
| Counts, DE genes in gene set; Size, total genes in gene set; Up, up-regulated genes; Dn, down-regulated genes. | | | | | | | |
