# Supplementary material for: Brain Transcriptomic Response to Social Eavesdropping in Zebrafish (Danio rerio)
Source: PLoS One. 2015 Dec 29;10(12):e0145801. doi: 10.1371/journal.pone.0145801 (PMC4700982; doi:10.1371/journal.pone.0145801)
Supplement: S5 Table — BINIC had no gene set over-represented. Gene sets list sorted by P-value. (DOC) [file pone.0145801.s008.doc]

**S5 Table.** Wikipathway gene sets over-represented in the differentially expressed genes [*P*-value < 0.1] for bystanders to interacting conspecifics (BIC), bystanders attentive to non-interacting conspecifics (BANIC) and bystanders inattentive to non-interacting conspecifics (BINIC). BINIC had no gene set over-represented. Gene sets list sorted by *P*-value.

| Group | ID | Description | *P*-value | Counts | Size | Up | Dn |
| --- | --- | --- | --- | --- | --- | --- | --- |
| BIC | WP1337 | **MAPK signaling pathway** | 0.00249 | 2 | 132 | 2 | 0 |
|  | WP1372 | Oxidative Stress | 0.01921 | 1 | 22 | 1 | 0 |
|  | WP444 | Signaling of Hepatocyte Growth Factor Receptor | 0.02345 | 1 | 33 | 1 | 0 |
|  | WP1385 | Nuclear Receptors | 0.02345 | 1 | 29 | 1 | 0 |
|  | WP1358 | Selenium metabolism Selenoproteins | 0.02769 | 1 | 36 | 1 | 0 |
|  | WP230 | TGF Beta Signaling Pathway | 0.03402 | 1 | 46 | 1 | 0 |
|  | WP1330 | Estrogen Signaling | 0.03823 | 1 | 70 | 1 | 0 |
|  | WP1384 | Toll-like receptor signaling pathway | 0.04663 | 1 | 65 | 1 | 0 |
|  | WP1322 | IL-6 Signaling Pathway | 0.05081 | 1 | 92 | 1 | 0 |
|  | WP1345 | T Cell Receptor Signaling Pathway | 0.08805 | 1 | 100 | 1 | 0 |
|  | WP1367 | TGF-beta Receptor Signaling Pathway | 0.08805 | 1 | 142 | 1 | 0 |
|  | WP1313 | Insulin Signaling | 0.09010 | 1 | 141 | 1 | 0 |
|  | WP1323 | EGFR1 Signaling Pathway | 0.09623 | 1 | 153 | 1 | 0 |
| BANIC | WP1337 | **MAPK signaling pathway** | 0.00249 | 2 | 132 | 2 | 0 |
|  | WP1372 | Oxidative Stress | 0.01921 | 1 | 22 | 1 | 0 |
|  | WP444 | Signaling of Hepatocyte Growth Factor Receptor | 0.02345 | 1 | 33 | 1 | 0 |
|  | WP1385 | Nuclear Receptors | 0.02345 | 1 | 29 | 1 | 0 |
|  | WP1358 | Selenium metabolism Selenoproteins | 0.02769 | 1 | 36 | 1 | 0 |
|  | WP230 | TGF Beta Signaling Pathway | 0.03402 | 1 | 46 | 1 | 0 |
|  | WP1330 | Estrogen Signaling | 0.03823 | 1 | 70 | 1 | 0 |
|  | WP1384 | Toll-like receptor signaling pathway | 0.04663 | 1 | 65 | 1 | 0 |
|  | WP1322 | IL-6 Signaling Pathway | 0.05081 | 1 | 92 | 1 | 0 |
|  | WP1345 | T Cell Receptor Signaling Pathway | 0.08805 | 1 | 100 | 1 | 0 |
|  | WP1367 | TGF-beta Receptor Signaling Pathway | 0.08805 | 1 | 142 | 1 | 0 |
|  | WP1313 | Insulin Signaling | 0.09010 | 1 | 141 | 1 | 0 |
|  | WP1323 | EGFR1 Signaling Pathway | 0.09623 | 1 | 153 | 1 | 0 |
| Counts, DE genes in gene set; Size, total genes in gene set; Up, up-regulated genes; Dn, down-regulated genes. | | | | | | | |
