# Supplementary material for: Brain Transcriptomic Response to Social Eavesdropping in Zebrafish (Danio rerio)
Source: PLoS One. 2015 Dec 29;10(12):e0145801. doi: 10.1371/journal.pone.0145801 (PMC4700982; doi:10.1371/journal.pone.0145801)
Supplement: S6 Table — Gene sets list sorted by P-value. (DOC) [file pone.0145801.s009.doc]

**S6 Table.** GO Biological process gene sets over-represented in the differentially expressed genes [*P*-value < 0.1] for bystanders to interacting conspecifics (BIC), bystanders attentive to non-interacting conspecifics (BANIC) and bystanders inattentive to non-interacting conspecifics (BINIC). Gene sets list sorted by *P*-value.

| Group | ID | Description | *P*-value | Counts | Size | Up | Dn |
| --- | --- | --- | --- | --- | --- | --- | --- |
| BIC | GO:0051252 | regulation of RNA metabolic process | 0.00690 | 3 | 551 | 3 | 0 |
|  | GO:0032774 | RNA biosynthetic process | 0.00719 | 3 | 559 | 3 | 0 |
|  | GO:0010556 | regulation of macromolecule biosynthetic process | 0.00798 | 3 | 580 | 3 | 0 |
|  | GO:0031326 | regulation of cellular biosynthetic process | 0.00858 | 3 | 595 | 3 | 0 |
|  | GO:0051171 | regulation of nitrogen compound metabolic process | 0.00917 | 3 | 609 | 3 | 0 |
|  | GO:0019438 | aromatic compound biosynthetic process | 0.01232 | 3 | 676 | 3 | 0 |
|  | GO:0044271 | cellular nitrogen compound biosynthetic process | 0.01248 | 3 | 679 | 3 | 0 |
|  | GO:0018130 | heterocycle biosynthetic process | 0.01258 | 3 | 681 | 3 | 0 |
|  | GO:0080090 | regulation of primary metabolic process | 0.01354 | 3 | 699 | 3 | 0 |
|  | GO:1901362 | organic cyclic compound biosynthetic process | 0.01392 | 3 | 706 | 3 | 0 |
|  | GO:0034645 | cellular macromolecule biosynthetic process | 0.01705 | 3 | 759 | 3 | 0 |
|  | GO:0010467 | gene expression | 0.01762 | 3 | 768 | 3 | 0 |
|  | GO:0030522 | intracellular receptor signaling pathway | 0.02006 | 1 | 24 | 1 | 0 |
|  | GO:0090304 | nucleic acid metabolic process | 0.02108 | 3 | 819 | 3 | 0 |
|  | GO:0043401 | steroid hormone mediated signaling pathway | 0.02997 | 1 | 36 | 1 | 0 |
|  | GO:0006355 | regulation of transcription, DNA-dependent | 0.03201 | 2 | 450 | 2 | 0 |
|  | GO:0048545 | response to steroid hormone stimulus | 0.03735 | 1 | 45 | 1 | 0 |
|  | GO:0009058 | **biosynthetic process** | 0.03748 | 3 | 1010 | 3 | 0 |
|  | GO:0071407 | cellular response to organic cyclic compound | 0.03980 | 1 | 48 | 1 | 0 |
|  | GO:0006457 | protein folding | 0.04061 | 1 | 49 | 1 | 0 |
|  | GO:0071396 | cellular response to lipid | 0.04061 | 1 | 49 | 1 | 0 |
|  | GO:0032870 | cellular response to hormone stimulus | 0.04305 | 1 | 52 | 1 | 0 |
|  | GO:0044237 | **cellular metabolic process** | 0.05579 | 4 | 2097 | 4 | 0 |
|  | GO:0009719 | response to endogenous stimulus | 0.06880 | 1 | 84 | 1 | 0 |
|  | GO:0006357 | regulation of transcription from RNA polymerase II promoter | 0.07752 | 1 | 95 | 1 | 0 |
|  | GO:0071704 | organic substance metabolic process | 0.08677 | 4 | 2373 | 4 | 0 |
| BANIC | GO:0051252 | regulation of RNA metabolic process | 0.01285 | 3 | 551 | 3 | 0 |
|  | GO:0032774 | RNA biosynthetic process | 0.01337 | 3 | 559 | 3 | 0 |
|  | GO:0010556 | regulation of macromolecule biosynthetic process | 0.01481 | 3 | 580 | 3 | 0 |
|  | GO:0031326 | regulation of cellular biosynthetic process | 0.01590 | 3 | 595 | 3 | 0 |
|  | GO:0051171 | regulation of nitrogen compound metabolic process | 0.01695 | 3 | 609 | 3 | 0 |
|  | GO:0019438 | aromatic compound biosynthetic process | 0.02257 | 3 | 676 | 3 | 0 |
|  | GO:0044271 | cellular nitrogen compound biosynthetic process | 0.02285 | 3 | 679 | 3 | 0 |
|  | GO:0018130 | heterocycle biosynthetic process | 0.02303 | 3 | 681 | 3 | 0 |
|  | GO:0030522 | intracellular receptor signaling pathway | 0.02402 | 1 | 24 | 1 | 0 |
|  | GO:0080090 | regulation of primary metabolic process | 0.02473 | 3 | 699 | 3 | 0 |
|  | GO:1901362 | organic cyclic compound biosynthetic process | 0.02540 | 3 | 706 | 3 | 0 |
|  | GO:0034645 | cellular macromolecule biosynthetic process | 0.03089 | 3 | 759 | 3 | 0 |
|  | GO:0010467 | gene expression | 0.03189 | 3 | 768 | 3 | 0 |
|  | GO:0043401 | steroid hormone mediated signaling pathway | 0.03586 | 1 | 36 | 1 | 0 |
|  | GO:0090304 | nucleic acid metabolic process | 0.03787 | 3 | 819 | 3 | 0 |
|  | GO:0048545 | response to steroid hormone stimulus | 0.04465 | 1 | 45 | 1 | 0 |
|  | GO:0071407 | cellular response to organic cyclic compound | 0.04757 | 1 | 48 | 1 | 0 |
|  | GO:0071396 | cellular response to lipid | 0.04854 | 1 | 49 | 1 | 0 |
|  | GO:0006355 | regulation of transcription, DNA-dependent | 0.05065 | 2 | 450 | 2 | 0 |
|  | GO:0032870 | cellular response to hormone stimulus | 0.05144 | 1 | 52 | 1 | 0 |
|  | GO:0007156 | homophilic cell adhesion | 0.05338 | 1 | 54 | 1 | 0 |
|  | GO:0009058 | **biosynthetic process** | 0.06560 | 3 | 1010 | 3 | 0 |
|  | GO:0009719 | response to endogenous stimulus | 0.08199 | 1 | 84 | 1 | 0 |
|  | GO:0006357 | regulation of transcription from RNA polymerase II promoter | 0.09230 | 1 | 95 | 1 | 0 |
| BINIC | GO:0007156 | homophilic cell adhesion | 0.00910 | 1 | 54 | 1 | 0 |
|  | GO:0007155 | cell adhesion | 0.02089 | 1 | 124 | 1 | 0 |
| Counts, DE genes in gene set; Size, total genes in gene set; Up, up-regulated genes; Dn, down-regulated genes. | | | | | | | |
