# Supplementary material for: Brain Transcriptomic Response to Social Eavesdropping in Zebrafish (Danio rerio)
Source: PLoS One. 2015 Dec 29;10(12):e0145801. doi: 10.1371/journal.pone.0145801 (PMC4700982; doi:10.1371/journal.pone.0145801)
Supplement: S7 Table — Gene sets list sorted by P-value. (DOC) [file pone.0145801.s010.doc]

**S7 table.** GO Cellular component gene sets over-represented in the differentially expressed genes [*P*-value < 0.1] for bystanders to interacting conspecifics (BIC), bystanders attentive to non-interacting conspecifics (BANIC) and bystanders inattentive to non-interacting conspecifics (BINIC) Gene sets list sorted by *P*-value.

| Group | ID | Description | *P*-value | Counts | Size | Up | Dn |
| --- | --- | --- | --- | --- | --- | --- | --- |
| BIC | GO:0005634 | **nucleus** | 0.01578 | 3 | 879 | 3 | 0 |
| BANIC | GO:0005634 | **nucleus** | 0.06083 | 3 | 879 | 3 | 0 |
|  | GO:0044464 | cell part | 0.09602 | 5 | 2581 | 4 | 1 |
| BINIC | GO:0005886 | plasma membrane | 0.09005 | 1 | 479 | 1 | 0 |
| Counts, DE genes in gene set; Size, total genes in gene set; Up, up-regulated genes; Dn, down-regulated genes. | | | | | | | |
