# Supplementary material for: Brain Transcriptomic Response to Social Eavesdropping in Zebrafish (Danio rerio)
Source: PLoS One. 2015 Dec 29;10(12):e0145801. doi: 10.1371/journal.pone.0145801 (PMC4700982; doi:10.1371/journal.pone.0145801)
Supplement: S8 Table — Gene sets list sorted by P-value. (DOC) [file pone.0145801.s011.doc]

**S8 Table.** GO Molecular function gene sets over-represented in the differentially expressed genes [*P*-value < 0.1] for bystanders to interacting conspecifics (BIC), bystanders attentive to non-interacting conspecifics (BANIC) and bystanders inattentive to non-interacting conspecifics (BINIC). Gene sets list sorted by *P*-value.

| Group | ID | Description | *P*-value | Counts | Size | Up | Dn |
| --- | --- | --- | --- | --- | --- | --- | --- |
| BIC | GO:0003676 | nucleic acid binding | 0.00343 | 4 | 1056 | 4 | 0 |
|  | GO:0004879 | ligand-activated sequence-specific DNA binding RNA polymerase II transcription factor activity | 0.01502 | 1 | 19 | 1 | 0 |
|  | GO:0051082 | unfolded protein binding | 0.01816 | 1 | 23 | 1 | 0 |
|  | GO:0043565 | sequence-specific DNA binding | 0.02140 | 2 | 306 | 2 | 0 |
|  | GO:0003690 | double-stranded DNA binding | 0.02519 | 1 | 32 | 1 | 0 |
|  | GO:0003707 | steroid hormone receptor activity | 0.02830 | 1 | 36 | 1 | 0 |
|  | GO:0003700 | sequence-specific DNA binding transcription factor activity | 0.03176 | 2 | 377 | 2 | 0 |
|  | GO:0005488 | **binding** | 0.04302 | 5 | 3353 | 5 | 0 |
|  | GO:0004871 | signal transducer activity | 0.06410 | 2 | 551 | 2 | 0 |
|  | GO:0046983 | protein dimerization activity | 0.09700 | 1 | 127 | 1 | 0 |
| BANIC | GO:0003676 | nucleic acid binding | 0.01804 | 4 | 1056 | 4 | 0 |
|  | GO:0004879 | ligand-activated sequence-specific DNA binding RNA polymerase II transcription factor activity | 0.02097 | 1 | 19 | 1 | 0 |
|  | GO:0003690 | double-stranded DNA binding | 0.03510 | 1 | 32 | 1 | 0 |
|  | GO:0046872 | metal ion binding | 0.03696 | 4 | 1297 | 3 | 1 |
|  | GO:0003707 | steroid hormone receptor activity | 0.03941 | 1 | 36 | 1 | 0 |
|  | GO:0043565 | sequence-specific DNA binding | 0.04213 | 2 | 306 | 2 | 0 |
|  | GO:0003700 | sequence-specific DNA binding transcription factor activity | 0.06158 | 2 | 377 | 2 | 0 |
|  | GO:0008270 | zinc ion binding | 0.07125 | 2 | 409 | 1 | 1 |
|  | GO:0005488 | **binding** | 0.08718 | 6 | 3353 | 5 | 1 |
| BINIC | GO:0005509 | calcium ion binding | 0.04214 | 1 | 265 | 1 | 0 |
| Counts, DE genes in gene set; Size, total genes in gene set; Up, up-regulated genes; Dn, down-regulated genes. | | | | | | | |
