# Supplementary material for: Brain Transcriptomic Response to Social Eavesdropping in Zebrafish (Danio rerio)
Source: PLoS One. 2015 Dec 29;10(12):e0145801. doi: 10.1371/journal.pone.0145801 (PMC4700982; doi:10.1371/journal.pone.0145801)
Supplement: S9 Table — Gene sets list sorted by P-value. (DOC) [file pone.0145801.s012.doc]

**S9 Table.** Chromosome over-represented in the differentially expressed genes [*P*-value < 0.1] for bystanders to interacting conspecifics (BIC), bystanders attentive to non-interacting conspecifics (BANIC) and bystanders inattentive to non-interacting conspecifics (BINIC). Gene sets list sorted by *P*-value.

| Group | ID | Description | *P*-value | Counts | Size | Up | Dn |
| --- | --- | --- | --- | --- | --- | --- | --- |
| BIC | 23 | **Chromosome 23** | 0.026257 | 2 | 1216 | 2 | 0 |
|  | 21 | Chromosome 21 | 0.033954 | 2 | 1237 | 2 | 0 |
| BANIC | 23 | **Chromosome 23** | 0.004719 | 3 | 1216 | 2 | 1 |
|  | 14 | **Chromosome 14** | 0.006025 | 3 | 1201 | 3 | 0 |
| BINIC | 14 | **Chromosome 14** | 0.003728 | 2 | 1201 | 2 | 0 |
| Counts, DE genes in gene set; Size, total genes in gene set; Up, up-regulated genes; Dn, down-regulated genes. | | | | | | | |
