# Supplementary material for: Brain Transcriptomic Response to Social Eavesdropping in Zebrafish (Danio rerio)
Source: PLoS One. 2015 Dec 29;10(12):e0145801. doi: 10.1371/journal.pone.0145801 (PMC4700982; doi:10.1371/journal.pone.0145801)
Supplement: S10 Table — Gene sets list sorted by P-value. (DOC) [file pone.0145801.s013.doc]

**S10 Table.** KEGG gene sets differentially expressed considering under- and over-expressed genes [*P*-value < 0.1] for bystanders to interacting conspecifics (BIC), bystanders attentive to non-interacting conspecifics (BANIC) and bystanders inattentive to non-interacting conspecifics (BINIC). Gene sets list sorted by *P*-value.

| Group | ID | Description | *P*-value | FDR | Size |
| --- | --- | --- | --- | --- | --- |
| BIC | 4744 | **Phototransduction** | 0.001 | 0.096 | 23 |
|  | 4710 | **Circadian rhythm - mammal** | 0.049 | 0.936 | 15 |
|  | 4010 | **MAPK signaling pathway** | 0.051 | 0.936 | 107 |
|  | 100 | **Steroid biosynthesis** | 0.086 | 0.936 | 18 |
| BANIC | 190 | **Oxidative phosphorylation** | 0.041 | 0.905 | 48 |
| BINIC | 190 | **Oxidative phosphorylation** | 0.006 | 0.421 | 48 |
|  | 3010 | Ribosome | 0.008 | 0.421 | 34 |
| FDR, false discovery rate. | | | | | |
