# Supplementary material for: Brain Transcriptomic Response to Social Eavesdropping in Zebrafish (Danio rerio)
Source: PLoS One. 2015 Dec 29;10(12):e0145801. doi: 10.1371/journal.pone.0145801 (PMC4700982; doi:10.1371/journal.pone.0145801)
Supplement: S11 Table — Gene sets list sorted by P-value. (DOC) [file pone.0145801.s014.doc]

**S11 Table.** Wikipathway gene sets differentially expressed considering under- and over-expressed genes [*P*-value < 0.1] for bystanders to interacting conspecifics (BIC), bystanders attentive to non-interacting conspecifics (BANIC) and bystanders inattentive to non-interacting conspecifics (BINIC). Gene sets list sorted by *P*-value.

| Group | ID | Description | *P*-value | FDR | Size |
| --- | --- | --- | --- | --- | --- |
| BFC | WP1387 | **Cholesterol Biosynthesis** | 0.017 | 0.637 | 13 |
|  | WP152 | **FGF signaling pathway** | 0.021 | 0.637 | 56 |
|  | WP402 | **ERK1 - ERK2 MAPK cascade** | 0.057 | 0.700 | 59 |
|  | WP1351 | Apoptosis | 0.061 | 0.700 | 30 |
|  | WP1367 | **TGF-beta Receptor Signaling Pathway** | 0.063 | 0.700 | 42 |
|  | WP1337 | **MAPK signaling pathway** | 0.069 | 0.700 | 48 |
|  | WP562 | **Exercise-induced Circadian Regulation** | 0.079 | 0.700 | 22 |
| BANC | WP1335 | **Oxidative phosphorylation** | 0.080 | 0.818 | 17 |
|  | WP152 | **FGF signaling pathway** | 0.089 | 0.818 | 56 |
| BINC | WP1387 | **Cholesterol Biosynthesis** | 0.002 | 0.057 | 13 |
|  | WP1335 | **Oxidative phosphorylation** | 0.003 | 0.057 | 17 |
|  | WP1339 | **Electron Transport Chain** | 0.003 | 0.057 | 32 |
|  | WP324 | Cytoplasmic Ribosomal Proteins | 0.005 | 0.084 | 29 |
| FDR, false discovery rate. | | | | | |
