# Supplementary material for: Brain Transcriptomic Response to Social Eavesdropping in Zebrafish (Danio rerio)
Source: PLoS One. 2015 Dec 29;10(12):e0145801. doi: 10.1371/journal.pone.0145801 (PMC4700982; doi:10.1371/journal.pone.0145801)
Supplement: S12 Table — Gene sets list sorted by P-value. (DOC) [file pone.0145801.s015.doc]

**S12 Table.** GO Biological process gene sets differentially expressed considering only over-expressed genes [*P*-value < 0.1] for bystanders to interacting conspecifics (BIC), bystanders attentive to non-interacting conspecifics (BANIC) and bystanders inattentive to non-interacting conspecifics (BINIC). Gene sets list sorted by *P*-value.

| Group | ID | Description | *P*-value | FDR | Size |
| --- | --- | --- | --- | --- | --- |
| BIC | GO:0040007 | **growth** | 0.022 | 0.831 | 23 |
|  | GO:0006351 | **transcription, DNA-templated** | 0.043 | 0.831 | 297 |
|  | GO:0006950 | **response to stress** | 0.051 | 0.831 | 43 |
|  | GO:0006629 | **lipid metabolic process** | 0.058 | 0.831 | 74 |
|  | GO:0007264 | small GTPase mediated signal transduction | 0.059 | 0.831 | 96 |
|  | GO:0006139 | nucleobase-containing compound metabolic process | 0.077 | 0.831 | 14 |
|  | GO:0001706 | endoderm formation | 0.085 | 0.831 | 10 |
|  | GO:0009790 | embryo development | 0.087 | 0.831 | 61 |
| BANIC | GO:0042742 | defense response to bacterium | 0.029 | 0.911 | 14 |
|  | GO:0001568 | blood vessel development | 0.037 | 0.911 | 23 |
|  | GO:0006508 | proteolysis | 0.045 | 0.911 | 231 |
|  | GO:0006813 | potassium ion transport | 0.055 | 0.911 | 29 |
|  | GO:0008033 | **tRNA processing** | 0.069 | 0.911 | 24 |
|  | GO:0045454 | cell redox homeostasis | 0.069 | 0.911 | 20 |
|  | GO:0007169 | **transmembrane receptor protein tyrosine kinase signaling pathway** | 0.070 | 0.911 | 21 |
|  | GO:0007219 | **Notch signaling pathway** | 0.070 | 0.911 | 20 |
|  | GO:0034765 | regulation of ion transmembrane transport | 0.082 | 0.911 | 34 |
|  | GO:0048514 | blood vessel morphogenesis | 0.084 | 0.911 | 12 |
|  | GO:0007601 | **visual perception** | 0.088 | 0.911 | 45 |
|  | GO:0043049 | **otic placode formation** | 0.089 | 0.911 | 17 |
| BINIC | GO:0007010 | cytoskeleton organization | 0.022 | 0.921 | 11 |
|  | GO:0001568 | blood vessel development | 0.037 | 0.921 | 23 |
|  | GO:0008033 | **tRNA processing** | 0.047 | 0.921 | 24 |
|  | GO:0006396 | **RNA processing** | 0.057 | 0.921 | 11 |
|  | GO:0070654 | **sensory epithelium regeneration** | 0.058 | 0.921 | 10 |
|  | GO:0048514 | blood vessel morphogenesis | 0.074 | 0.921 | 12 |
|  | GO:0042742 | defense response to bacterium | 0.081 | 0.921 | 14 |
|  | GO:0043066 | **negative regulation of apoptotic process** | 0.093 | 0.921 | 30 |
|  | GO:0045893 | **positive regulation of transcription, DNA-templated** | 0.098 | 0.921 | 28 |
|  | GO:0006813 | potassium ion transport | 0.100 | 0.921 | 29 |
| FDR, false discovery rate. | | | | | |
