# Supplementary material for: Brain Transcriptomic Response to Social Eavesdropping in Zebrafish (Danio rerio)
Source: PLoS One. 2015 Dec 29;10(12):e0145801. doi: 10.1371/journal.pone.0145801 (PMC4700982; doi:10.1371/journal.pone.0145801)
Supplement: S13 Table — Gene sets list sorted by P-value. (DOC) [file pone.0145801.s016.doc]

**S13 Table.** GO Cellular Compartment process gene sets differentially expressed considering only over-expressed genes [*P*-value < 0.1] for bystanders to interacting conspecifics (BIC), bystanders attentive to non-interacting conspecifics (BANIC) and bystanders inattentive to non-interacting conspecifics (BINIC). Gene sets list sorted by *P*-value.

| Group | ID | Description | *P*-value | FDR | Size |
| --- | --- | --- | --- | --- | --- |
| BIC | GO:0005743 | mitochondrial inner membrane | 0.021 | 0.864 | 39 |
|  | GO:0005622 | intracellular | 0.078 | 0.864 | 263 |
|  | GO:0005576 | **extracellular region** | 0.081 | 0.864 | 322 |
|  | GO:0008305 | **integrin complex** | 0.084 | 0.864 | 16 |
| BANIC | GO:0005777 | **peroxisome** | 0.032 | 0.816 | 11 |
| BINIC | GO:0005777 | **peroxisome** | 0.056 | 0.922 | 11 |
| FDR, false discovery rate. | | | | | |
