# Supplementary material for: Brain Transcriptomic Response to Social Eavesdropping in Zebrafish (Danio rerio)
Source: PLoS One. 2015 Dec 29;10(12):e0145801. doi: 10.1371/journal.pone.0145801 (PMC4700982; doi:10.1371/journal.pone.0145801)
Supplement: S14 Table — Gene sets list sorted by P-value. (DOC) [file pone.0145801.s017.doc]

**S14 Table.** GO Molecular Function process gene sets differentially expressed considering only over-expressed genes [*P*-value < 0.1] for bystanders to interacting conspecifics (BIC), bystanders attentive to non-interacting conspecifics (BANIC) and bystanders inattentive to non-interacting conspecifics (BINIC). Gene sets list sorted by *P*-value.

| Group | ID | Description | *P*-value | FDR | Size |
| --- | --- | --- | --- | --- | --- |
| BIC | GO:0003700 | **sequence-specific DNA binding transcription factor activity** | 0.025 | 0.939 | 391 |
|  | GO:0003735 | **structural constituent of ribosome** | 0.049 | 0.939 | 53 |
|  | GO:0004129 | **cytochrome-c oxidase activity** | 0.055 | 0.939 | 10 |
|  | GO:0019829 | **cation-transporting ATPase activity** | 0.057 | 0.939 | 13 |
|  | GO:0017017 | **MAP kinase tyrosine/serine/threonine phosphatase activity** | 0.063 | 0.939 | 15 |
|  | GO:0005525 | **GTP binding** | 0.069 | 0.939 | 201 |
|  | GO:0004364 | **glutathione transferase activity** | 0.076 | 0.939 | 12 |
|  | GO:0005520 | insulin-like growth factor binding | 0.076 | 0.939 | 13 |
|  | GO:0004725 | **protein tyrosine phosphatase activity** | 0.082 | 0.939 | 44 |
|  | GO:0004935 | **adrenergic receptor activity** | 0.083 | 0.939 | 14 |
|  | GO:0008134 | **transcription factor binding** | 0.090 | 0.939 | 28 |
| BANIC | GO:0031418 | L-ascorbic acid binding | 0.029 | 0.890 | 13 |
|  | GO:0004896 | cytokine receptor activity | 0.046 | 0.890 | 12 |
|  | GO:0004252 | serine-type endopeptidase activity | 0.050 | 0.890 | 84 |
|  | GO:0004842 | ubiquitin-protein transferase activity | 0.058 | 0.890 | 29 |
|  | GO:0003723 | **RNA binding** | 0.061 | 0.890 | 107 |
|  | GO:0004714 | **transmembrane receptor protein tyrosine kinase activity** | 0.068 | 0.890 | 17 |
|  | GO:0016798 | hydrolase activity, acting on glycosyl bonds | 0.084 | 0.890 | 20 |
|  | GO:0000975 | **regulatory region DNA binding** | 0.089 | 0.890 | 12 |
|  | GO:0005249 | **voltage-gated potassium channel activity** | 0.091 | 0.890 | 18 |
|  | GO:0009881 | **photoreceptor activity** | 0.098 | 0.890 | 25 |
| BINIC | GO:0000975 | **regulatory region DNA binding** | 0.023 | 0.904 | 12 |
|  | GO:0008236 | serine-type peptidase activity | 0.043 | 0.904 | 53 |
|  | GO:0016798 | hydrolase activity, acting on glycosyl bonds | 0.068 | 0.904 | 20 |
|  | GO:0004518 | nuclease activity | 0.084 | 0.904 | 36 |
|  | GO:0003723 | **RNA binding** | 0.099 | 0.904 | 107 |
| FDR, false discovery rate. | | | | | |
