# Supplementary material for: Brain Transcriptomic Response to Social Eavesdropping in Zebrafish (Danio rerio)
Source: PLoS One. 2015 Dec 29;10(12):e0145801. doi: 10.1371/journal.pone.0145801 (PMC4700982; doi:10.1371/journal.pone.0145801)
Supplement: S15 Table — Gene sets list sorted by P-value. (DOC) [file pone.0145801.s018.doc]

**S15 Table.** Chromosome location gene sets differentially expressed considering under- and over-expressed genes (*P*-value < 0.1) for bystanders to interacting conspecifics (BIC), bystanders attentive to non-interacting conspecifics (BANIC) and bystanders inattentive to non-interacting conspecifics (BINIC). Gene sets list sorted by *P*-value.

| Group | ID | Description | *P*-value | FDR | Size |
| --- | --- | --- | --- | --- | --- |
| BIC | 14 | **Chromosome 14** | 0.010 | 0.238 | 382 |
|  | 24 | Chromosome 24 | 0.073 | 0.750 | 317 |
| BANIC | 14 | **Chromosome 14** | 0.000 | 0.000 | 382 |
|  | 11 | Chromosome 11 | 0.066 | 0.573 | 376 |
| BINIC | 14 | **Chromosome 14** | 0.000 | 0.001 | 382 |
| FDR, false discovery rate. | | | | | |
